# Radiation combined with immune checkpoint inhibitors for unresectable locally advanced non-small cell lung cancer: synergistic mechanisms, current state, challenges, and orientations

**DOI:** 10.1186/s12964-023-01139-8

**Published:** 2023-05-23

**Authors:** Leilei Wu, Zhenshan Zhang, Menglin Bai, Yujie Yan, Jinming Yu, Yaping Xu

**Affiliations:** 1 Department of Radiation Oncology, Shanghai Pulmonary Hospital, Tongji University School of Medicine, Shanghai, China; 2 Department of Radiation Oncology and Shandong Provincial Key Laboratory of Radiation Oncology, Shandong Cancer Hospital and Institute, Shandong First Medical University and Shandong Academy of Medical Sciences, Jinan, Shandong China; 3 Department of Radiation Oncology, Fudan University Shanghai Cancer Center, Shanghai, China; 4 Department of Radiation Oncology, Shanghai Proton and Heavy Ion Center, Fudan University Cancer Hospital, Shanghai, China

**Keywords:** Locally advanced non-small cell lung cancer (LA-NSCLC), Radiotherapy, Immune checkpoint inhibitors (ICIs), RT combined with ICIs (iRT), Advances, Challenges, Biomarkers

## Abstract

Until the advent of immune checkpoint inhibitors (ICIs), definitive radiotherapy (RT) concurrently with chemotherapy was recommended for unresectable, locally advanced non-small cell lung cancer (LA-NSCLC). The trimodality paradigm with consolidation ICIs following definitive concurrent chemoradiotherapy has been the standard of care since the PACIFIC trial. Preclinical evidence has demonstrated the role of RT in the cancer-immune cycle and the synergistic effect of RT combined with ICIs (iRT). However, RT exerts a double-edged effect on immunity and the combination strategy still could be optimized in many areas. In the context of LA-NSCLC, optimized RT modality, choice, timing, and duration of ICIs, care for oncogenic addicted tumors, patient selection, and novel combination strategies require further investigation. Targeting these blind spots, novel approaches are being investigated to cross the borders of PACIFIC. We discussed the development history of iRT and summarized the updated rationale for the synergistic effect. We then summarized the available research data on the efficacy and toxicity of iRT in LA-NSCLC for cross-trial comparisons to eliminate barriers. Progression during and after ICIs consolidation therapy has been regarded as a distinct resistance scenario from primary or secondary resistance to ICIs, the subsequent management of which has also been discussed. Finally, based on unmet needs, we probed into the challenges, strategies, and auspicious orientations to optimize iRT in LA-NSCLC. In this review, we focus on the underlying mechanisms and recent advances of iRT with an emphasis on future challenges and directions that warrant further investigation. Taken together, iRT is a proven and potential strategy in LA-NSCLC, with multiple promising approaches to further improve the efficacy.

Video Abstract

**Supplementary Information:**

The online version contains supplementary material available at 10.1186/s12964-023-01139-8.

## Background

Non-small cell lung cancer (NSCLC) accounts for approximately 85% of all lung cancer cases, and nearly one-third of patients have stage III, locally advanced (LA) disease at diagnosis [1]. Encompassing a heterogeneous group of tumor presentations, a multidisciplinary approach to define the resectability of stage III LA-NSCLC is mandatory [2]. Indeed, most LA-NSCLC patients lose the opportunity for curative resection at diagnosis. Radiotherapy (RT) has been used to cure malignant cancers in the past century, and approximately half of the cancer patients are treated with RT, which involves curative and palliative interventions [3]. The anti-tumor effects of RT have historically been regarded as radiation-induced deoxyribonucleic acid (DNA) deadly functional and structural changes that result in direct local cancerous cell apoptosis, senescence, and autophagy [3–5]. For more than two decades, the standard treatment for unresectable NSCLC has been thoracic RT [6].

Based on improved survival, definitive RT with concurrent platinum-based chemotherapy (concurrent chemoradiotherapy, cCRT) has become the standard of care (SoC) for unresectable LA-NSCLC [7, 8]. However, the outcome is still unsatisfactory, with the overall survival (OS) rate of 15–25% [2]. After the combination of targeted or chemotherapy consolidation treatment failed to bring survival benefits, the PACIFIC trial, like a huge "tsunami,” completely revolutionized the treatment of unresectable LA-NSCLC, accomplishing the success of RT combined with immune checkpoint inhibitors (ICIs) in stage III NSCLC [9]. ICIs combined with RT (immunoradiotherapy, iRT) have doubled the objective response rate (ORR) of advanced NSCLC in the PEMBRO-RT trial compared to ICI monotherapy [10]. It is believed that iRT can play a role of ‘1 + 1 > 2’ in treating tumors, for the synergistic anti-tumor effect of RT and immunotherapy. To date, a series of preclinical and clinical trials have been conducted to explore the theoretical basis and maximize the efficacy of iRT. Notably, for patients with unresectable LA-NSCLC, more novel patterns of combining RT and ICIs following the PACIFIC pattern are ongoing.

It is well known that RT should be administered in doses and fractionations suitable to ignite tumor-targeting immune responses, and innovative combination therapies can further improve outcomes in patients with unresectable LA-NSCLC. Herein, we review the underlying mechanisms and recent advances in iRT, summarize the current status and unmet needs of iRT in unresectable LA-NSCLC, and provide an overview of novel strategies for optimizing therapeutic effects.

### The history and development of iRT

Routinely, RT is a treatment against local lesions, bringing damage to both tumor cells and normal cells; research in this area has focused on the biological effects on tumor cells induced by RT. Accidently, a special “abscopal effect” was put forward by Mole in 1953 to describe the inhibition of metastatic diseases distant from the irradiated field [11]. In other words, apart from the role of local control, RT is also a weapon that provokes a systemic response. Naturally, changes in the surrounding stroma and tumor microenvironment (TME) triggered by damaged or necrotic tumor cells have gradually attracted the attention of researchers [12]. Meanwhile, efforts to eliminate resistance to RT have never ceased, which is closely related to the original TME and dynamic alterations in response to RT [13]. Moreover, in addition to novel RT modalities such as stereotactic body radiation therapy (SBRT) and charged particle RT, combining other treatments has been an effective strategy. For decades, RT has been successfully integrated with surgery, chemotherapy, and molecular-targeted therapy.

Antibodies against programmed cell death 1 (PD-1), programmed cell death-ligand 1 (PD-L1), and cytotoxic T-lymphocyte antigen 4 (CTLA-4) aim to block negative checkpoints of immune homeostasis and reinvigorate exhausted CD8 + T cells [14]. These ICIs have reshaped the treatment landscape of multiple solid tumors, including NSCLC [15]. Clearly, there is considerable interest in combination regimens of RT and ICIs [16]. PEMBRO-RT, the first phase II randomized trial in advanced NSCLC by Welsh et al., validated the safety of combining RT with pembrolizumab, but the benefits in survival deserve further exploration [10]. Subsequently, a pooled analysis of PEMBRO-RT and MDACC (phase I/II) by the team further demonstrated that pembrolizumab combined with RT significantly improved the efficacy and survival of patients with advanced NSCLC and confirmed that iRT enhanced the abscopal effect for the first time, raising the ORR of lesions out of the irradiated field from 19.7% to 41.7% [17]. In addition, a secondary analysis of the KEYNOTE-001 study also found that patients who had previously received RT achieved longer progression-free survival (PFS) and OS after treatment with pembrolizumab, with acceptable safety [18]. However, no large-scale phase III clinical studies have verified the efficacy of RT combined with ICIs in metastatic NSCLC, which requires further validation.

**Fig. 1:**
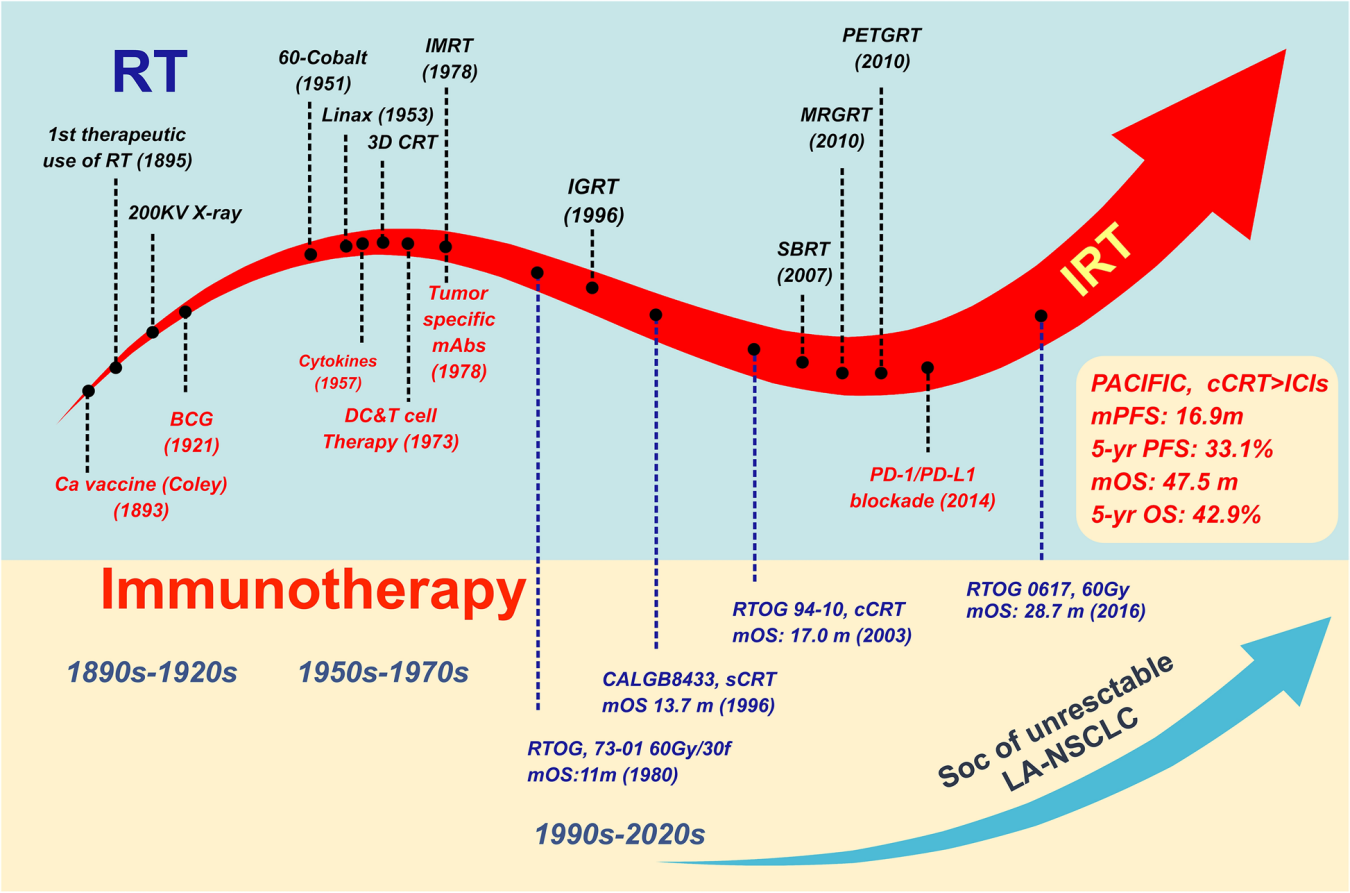
The history and development of iRT, along with shifts in SoC of unresectable LA-NSCLC. RT has experienced several technological revolutions though more than 100 years of development, from first use of radiation to to SBRT and image-guided RT. Cancer immunotherapy also has a history of more than 100 years, until PD-1/PD-L1 inhibitors were approved. On the other hand, RT has been the cornerstone of unresectable LA-NSCLC, while the amazing result from PACIFIC trial opened a new era of iRT in LA-NSCLC. Abbreviations: *RT* Radiotherapy, *ICIs* Immune checkpoint inhibitors, *PD-1* Programmed cell death 1, *PD-L1* Programmed cell death-ligand 1, *iRT* ICIs combined with RT, *SoC* Standard of care, *LA-NSCLC* Locally advanced non-small cell lung cancer, *cCRT* Concurrent chemoradiotherapy, *mPFS*, Median progression-free survival, *OS* Overall survival, *mOS* Median overall survival, *yr* Year, *SBRT* Stereotactic body radiation therapy

The PACIFIC trial verified the benefits of ICIs consolidation after definitive cCRT [9], initiating the application of iRT in LA-NSCLC  [8]. Of course, no matter what type of tumor and stage, to achieve an effect of “1 + 1 ≥ 2” of iRT, it is necessary to determine optimal dose and fractionation, irradiated site and field, RT modality, sequence of RT and ICIs, type of combined ICI, and suitable patients. In addition, attention should be paid to the safety of combined therapy, secondary resistance, and other promising combination strategies. We depict the history and development of iRT along with shifts in the SoC of unresectable LA-NSCLC in Fig. 1.

### Current cognitions of the effects of RT on immunity

#### RT participates in the cancer-immune cycle to exert a systemic anti-tumor effect

**Fig. 2:**
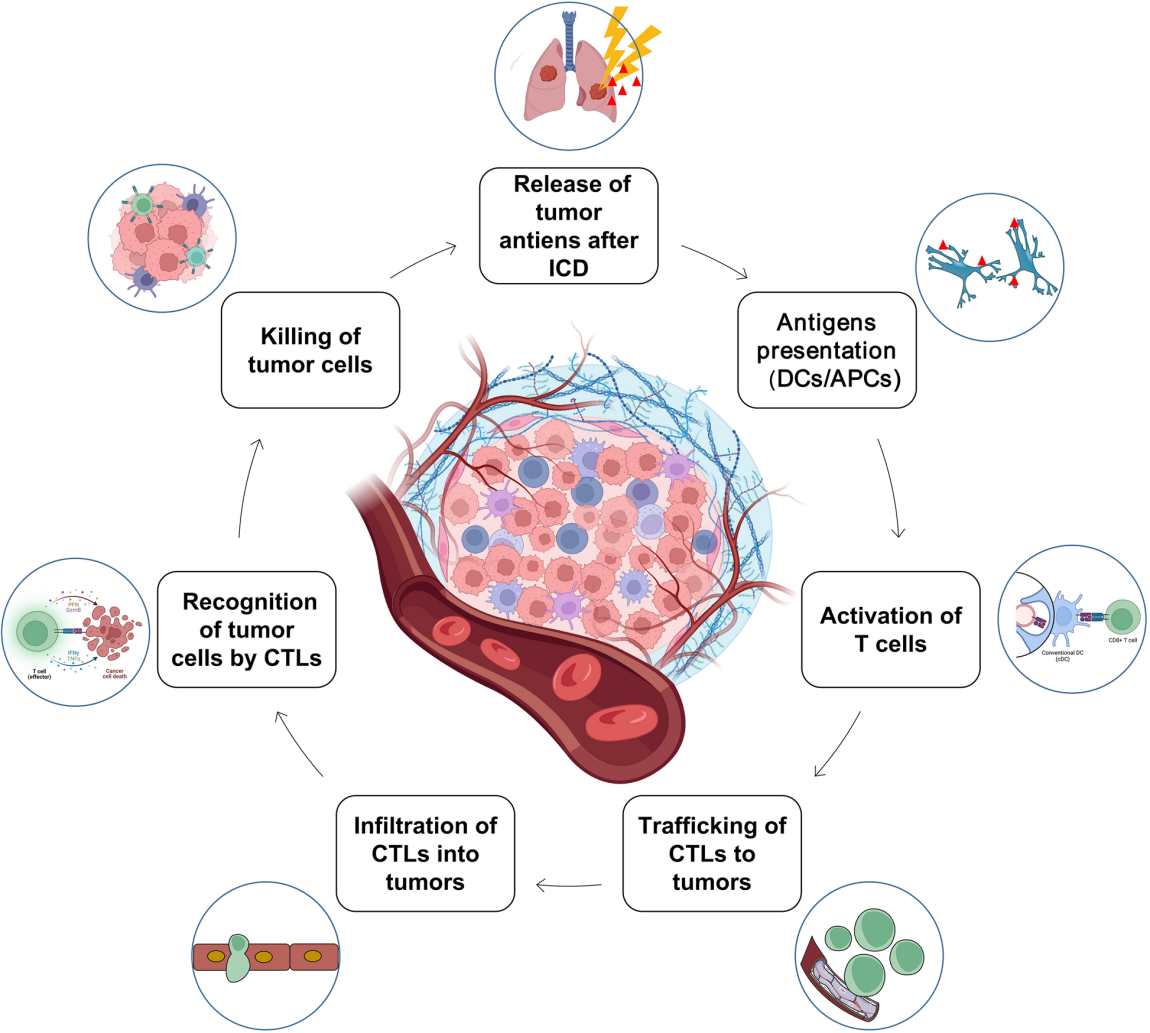
RT participates in the cancer-immune cycle. RT participates in the cancer-immune cycle, inducing a series of reactions for anti-tumor immunity activation. Abbreviations: *ICD* Immunogenic cell death, *DCs*, Dendritic cells, *APCs* Antigen-presenting cells, *CTLs* Cytotoxic lymphocytes

Although known for decades, abscopal responses induced by RT are rare and there is a lack of adequate explanation for this phenomenon. An estimated 46 cases of abscopal effects were reported between 1969 and 2014 [19]. A literature analysis reported that 7 (14%) cases of primary tumors and 41% of metastases were recorded in the lungs, among 51 cases of the abscopal effect at various locations [20]. After entering the era of immunotherapy, this phenomenon was confirmed to be mediated by immunity, as no abscopal effect was observed in immunodeficient mice [21]. RT is believed to induce a systemic, immune-mediated anti-tumor effect by participating in the cancer-immunity cycle, as illustrated in Fig. 2 In general, the series of reactions induced by RT provides a more supportive immune microenvironment for anti-tumor immunity, turning a “cold” tumor into a “hot” tumor, and the immunoregulatory effect of RT is exactly the theoretical basis of its abscopal effect [13].

### Essential immune-associated pathways activated by RT

RT activates multiple immune-associated pathways by damaging tumor cells, including cyclic guanosine monophosphate (GMP)-adenosine monophosphate (AMP) synthase (Cyclic GMP-AMP synthase, cGAS), and stimulator of interferon genes (STING) protein (cGAS-STING pathway) [22]. The cross-priming capacity of DCs induced by RT requires the activation of cGAS-STING and subsequent type I interferon signaling [23]. It is widely believed that the accumulation of cytoplasmic DNA, notably in the form of micronuclei, is essential for the cGAS-STING sensor [24]. Recently, Deng et al. found that released mitochondrial DNA (mtDNA), induced by the ZBP1-MLKL necroptotic signaling cascade, played a parallel role in triggering the cGAS-STING pathway in response to RT [25]. However, the latest research has also shown that cGAS-STING activation facilitated breast tumor progression in mouse xenograft models [26, 27], and knockdown of cGAS or STING expression prevents tumor metastasis [27]. Activation of cGAS-STING signaling may differentially affect diverse cell types in the TME, but how cGAS-STING activation mediates immunosuppression, conveying a tumor-promoting effect, remains poorly defined. Moreover, TLR 9, absent in melanoma 2 (AIM2), interferon-inducible protein 16 (IFI16), and others, could also sense the accumulation of DNA (extracellular or intracellular) and TLR 9 could heighten the downstream interferon regulatory factor (IRF) pathway, while AIM2 and IFI16 induced the secretion of inflammatory cytokines such as IL-1β and IL-18 [13].

### Double-edged sword function of RT on immunity

**Fig. 3:**
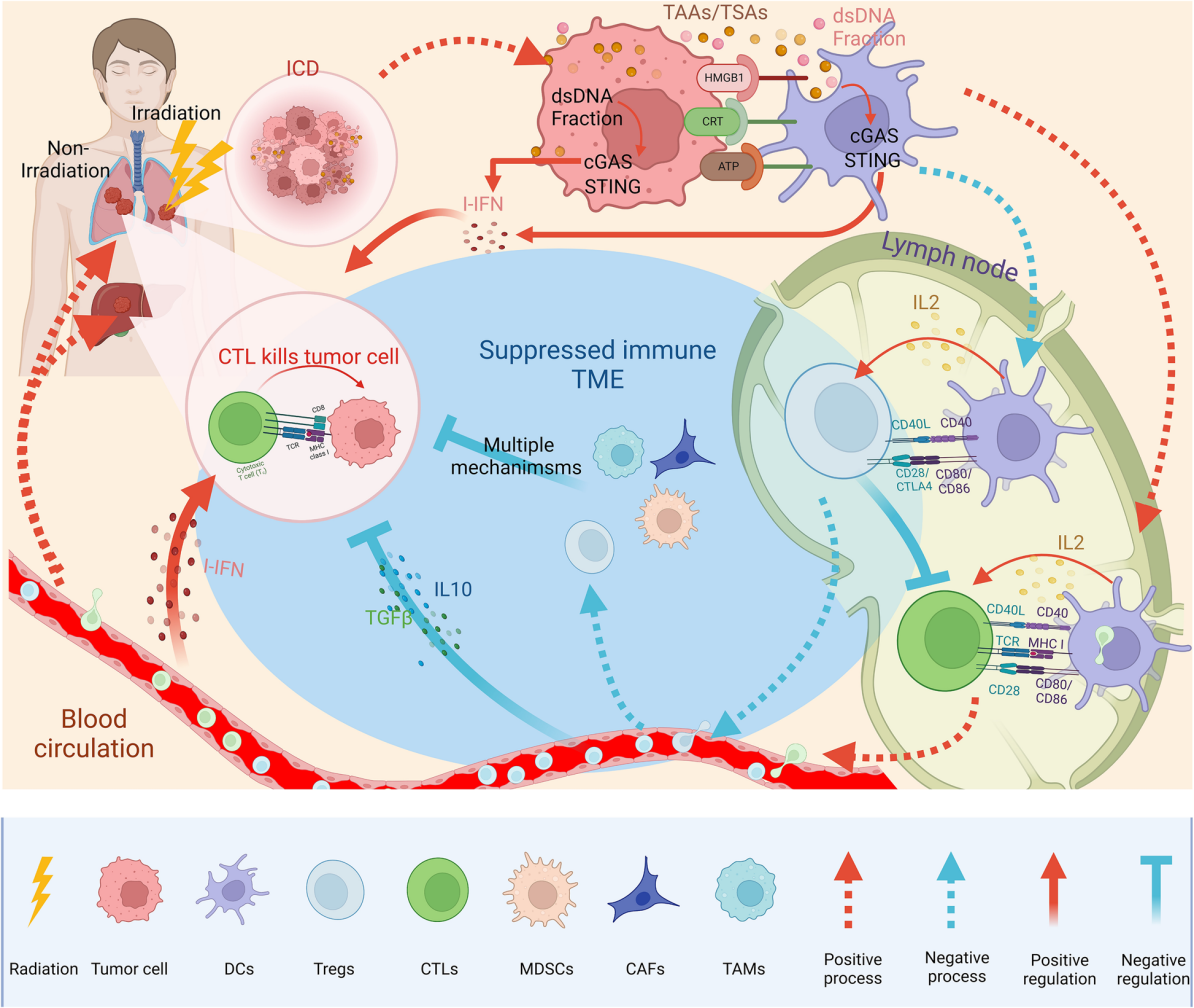
The double-edged sword immunomodulatory function of RT on TME. RT can exert a potent antitumor immune response by influencing almost all steps in the cancer-immunity cycle, from the first step of releasing antigens to the final immunomodulatory response. Some essential immune-associated pathways are activated by RT in the process, such as cGAS-STING pathway. However, RT may also induce a suppressed TME in the presence of overactivated MDSCs, TAMs, CAFs and Tregs. Such double-edged sword role would determing the final effect of combining RT and ICIs. Abbreviations: *RT* radiotherapy, *TME* Tumor microenvironment, *ICD* Immunogenic cell death, *GMP* Cyclic guanosine monophosphate, *AMP* Adenosine monophosphate, *cGAS* Cyclic GMP-AMP synthase, *STING* Stimulator of interferon genes, *MDSCs* Myeloid-derived suppressor cells, *DCs* Dendritic cells, *Tregs* The regulatory T cells, *CTLs* Cytotoxic lymphocytes, *TAMs* Tumor-associated macrophages, *CAFs* Cancer-associated fibroblasts

Both clinical and experimental observations suggest that RT may stimulate cancer cell metastasis and induce cancer-promoting effects. The double-edged sword immunomodulatory function of RT in TME is presented in Fig. 3. Myeloid-derived cells include tumor-associated macrophages (TAMs), DCs, polymorphonuclear neutrophils (PMNs), and myeloid-derived suppressor cells (MDSCs) [28, 29]. They are essential for facilitating anti-tumor immunity; however, a plastic immunomodulatory phenotype can be influenced by certain treatment factors. MDSCs are increased in the TME following RT in mouse models, the accumulation of which is through CCL2 – CCR2 signaling and CCL2 may be derived from tumor cells [30]. TAMs generally exhibit pro-tumor (M2-phenotype) properties, inducing angiogenesis and secreting immunosuppressive mediators such as IL-10 and TGF-β. They inhibit T cell function and anti-tumor immunity, and promote a radioresistant phenotype [31]. Stromal-derived factor 1 (SDF-1), colony stimulating factor-1 (CSF-1), and C-X-C chemokine receptor type 4 mediate the recruitment of TAMs and MDSCs, which are upregulated in the irradiated TME [32–34]. The regulatory T cells (Tregs) are also increased in the TME following RT, inducing immunosuppression by CTLA-4 expression, IL-10 release, and adenosine production by CD39 and CD73 ectonucleotidases [35]. Increased TGF-β and hypoxia-inducible factor 1-α (HIF-1α) levels can inhibit DCs maturation and induce radioresistance in endothelial cells. TGF-β can not only affect CD8 + T cell proliferation and function, but also induce CD4 + T cells to adopt a regulatory phenotype (Treg), thus dampening the radiation-induced anti-tumor immune response. Evidence indicates that hypofractionated radiation can result in a significant increase in TGF-β [36]. Typically, increased TAMs promote tumor growth, invasion, and metastasis by negatively regulating anti-tumor immunity, thus leading to worse tumor suppression [37]. Moreover, evidence has shown that SBRT with a single dose of 12 Gy or 15 Gy can result in the recruitment of CD4 + T cells mainly composed of Foxp3 + Tregs [36]. Furthermore, a study indicated that the increase in Tregs induced by radiation was dose-dependent, with a single dose of 20 Gy doubling that of a single dose of 2 Gy [38]. The accumulation of Tregs in the TME significantly abrogated anti-tumor immune responses, which confers surviving tumor cells with potent resistance to RT.

### The synergia of RT and ICIs

Most patients currently cannot benefit from ICIs, as they either do not respond to ICIs at all (innate or primary resistance) or acquire secondary resistance (acquired or secondary) after an initial period of response [39, 40]. Additionally, a fixed treatment duration for ICIs is being increasingly utilized in adjuvant and neoadjuvant settings. In parallel, the progression arising from resistance to ICIs during and after adjuvant therapy could have elements of either primary or secondary resistance, which is difficult to define. After considerable discussion, progression or resistance after treatment discontinuation for any reason has recently been distinguished from the above two resistance scenarios by the Society for Immunotherapy of Cancer [41]. The timeframe between the last dose of adjuvant therapy and disease progression further defined resistance in this setting. The taskforce ultimately agreed on 12 weeks as a cutoff to classify resistant disease in the adjuvant setting into “adequate treatment exposure” and “inadequate treatment exposure [41].”

The response rate is limited to 8–30% in unselective NSCLC patients with treatment of ICI monotherapy [42, 43]. RT participates in the cancer-immunity cycle by activating the immune system and providing a more supportive immune microenvironment for anti-tumor immunity, which alleviates primary resistance and delays the development of secondary resistance to ICIs. In addition, RT can be used to trigger an immune response after ICIs resistance. Elevated levels of PD-L1 can promote T cell exhaustion, a state characterized by dysfunction in T cell proliferation and effector function, related to immune escape and tolerance [44]. It has been observed in multiple cancer types that upregulated PD-L1 on tumor cells can be a dominant resistance mechanism to RT and CTLA4, demonstrating persistent T cell exhaustion and rapid progression [45]. Several studies have shown that PD-L1 upregulation can be detected after RT, which contributes to an explanation for tumor resistance to RT [23, 46, 47]. However, the addition of PD-L1 blockade can reverse T cell exhaustion to mitigate depression in the CD8-to-regulatory T cell ratio and further promote response and immunity through distinct mechanisms [23, 45, 47, 48]. Notably, it is generally accepted that as a leading biomarker, a relatively high expression of PD-L1 indicates a better response to ICIs, which also supports the use of ICIs to overcome resistance to RT [42, 43]. Thus, it can be assumed that PD-L1 upregulation induced by RT gives rise to an immunosuppressive tumor microenvironment, which, on the one hand, impairs the efficacy of RT but supports the use of ICIs in RT to overcome resistance. Similarly, the PEMBRO-RT trial showed that patients with low levels of PD-L1 expression benefit more from the combination of RT and ICIs in OS compared with patients with higher PD-L1 expression levels [10].

Many types of T cells, especially CD8 + T cells, which play a major role in the anti-tumor immune response, are activated and begin to reproduce via initiation of the cancer-immune cycle induced by RT. This assumption has been demonstrated in numerous studies and has been used to explain the abscopal effect of RT, a rare but promising phenomenon. Theoretically, the abscopal effect will be amplified with ICIs, which has also been shown in several preclinical studies [23, 25, 47]. The number of cases reporting abscopal effects at the beginning of the twentieth century increased significantly with the advent of immunotherapy [20]. Besides, the time of manifestation of the abscopal effect with regression of distant metastases was also reduced from 5.4 to 3.3 months, although statistically insignificant [20]. In addition, a study conducted by Deng et al. also found that RT alone cannot produce sustained anti-tumor immune effects, but a combination with PD-1/PD-L1 inhibitors can induce an increase in memory CD8 + T cells, resulting in long-lasting immune memory effects [23]. In general, RT combined with ICIs can overcome resistance to RT and ICIs, enhance the abscopal effect of RT, and strengthen the immune memory effect, generating a synergistic role in the anti-tumor response.

## IRT in unresectable LA-NSCLC

### Therapeutic efficacy

#### Consolidation ICIs after CRT

The traditional SoC for unresectable LA-NSCLC is definitive cCRT, with an unsatisfactory 5-year OS rate of 15%–25% [2]. However, the end of the patient’s survival curve was relatively flat, suggesting that there is still hope for a cure. After the combination of targeted or chemotherapy consolidation treatment failed to bring conspicuous survival benefits, the PACIFIC trial, like a huge “tsunami”, completely revolutionized the treatment of unresectable stage III NSCLC, and durvalumab as consolidation therapy for patients whose disease had not progressed after cCRT quickly became the new standard of care [9]. The last update with 5-year survival outcomes demonstrated that consolidation treatment with durvalumab brought robust and sustained OS and PFS benefits after cCRT compared with placebo, with median OS of 47.5 months (vs. 29.1) and 5-year OS rate of 42.9% (vs. 33.4%) [49]. In addition, the risk of death or distant metastasis was also reduced by 41% compared to placebo (stratified hazard ratio [HR], 0.59). However, an unplanned post hoc analysis showed that OS did not improve in tumors with PD-L1 expression ≤ 1% (HR, 1.15) [50]. The ongoing international retrospective PACIFIC-R study is assessing the real-world efficacy of durvalumab in patients from an early access program, which has also allowed sequential chemoradiotherapy (sCRT) in some countries [51]. The preliminary analysis of 1399 patients who received at least one cycle of durvalumab presented a median PFS of 21.7 months in the full population, with 23.7 months in patients treated with cCRT (77%) versus 19.3 months in patients treated with sCRT (14%). Notably, mirroring PACIFIC, the PFS was numerically longer among patients with PD-L1 expression ≥ 1% versus < 1% (22.4 vs. 15.6 months). LUN 14–179 is a phase II study aimed at evaluating the safety and efficacy of pembrolizumab as consolidation therapy after cCRT [52]. The results showed that consolidation pembrolizumab after cCRT prolonged the time to metastatic disease or death, PFS, and OS in comparison with historical controls of chemoradiotherapy (CRT) alone, and it did not increase the rates of grade 3–5 pneumonitis.

However, only half of the patients with stage III NSCLC are treated with radical intent in clinical practice, and only 2/3 receive cCRT [53]. Many patients are unable to tolerate cCRT owing to substantial toxicities and a high rate of treatment-related mortality [6, 54]. Thus, sCRT is widely used in clinical practice around the world as an option for patients who cannot tolerate or access cCRT. Whether PD-1 or PD-L1 inhibitors can prolong the survival of these patients is of great concern. GEMSTONE-301 is the first randomized, double-blind, multicenter, phase III trial to report adjuvant single-agent ICI for patients with stage III NSCLC whose disease had not progressed after sequential or concurrent CRT [55]. Updated data showed that the median PFS was significantly longer with sugemalimab as consolidation treatment than with placebo (10.5 vs. 6.2 months, *P* = 0.0012) [56]. The median PFS was 8.1 and 15.7 months in the sCRT and cCRT arms, respectively. The results demonstrated that sugemalimab is an effective consolidation therapy for patients with unresectable LA-NSCLC without disease progression after either cCRT or sCRT. In addition, the survival curves of PACIFIC-R for patients treated with sCRT also suggested a plateau and long-term benefit, similar to PACIFIC [51]. The primary safety and secondary efficacy analyses from the single-arm phase II open-label PACIFIC-6 trial were recently reported, and the primary endpoint was the incidence of grade ≥ 3 treatment-related adverse events (TRAEs) within 6 months [57]. Overall, 117 patients with ECOG PS ≤ 2 who did not have any progressive disease after sCRT received adjuvant durvalumab for up to 2 years. As a result, 22 (18.8%) patients developed grade 3 or 4 adverse events (AEs), and only 5 (4.3%) experienced grade 3 or 4 possibly related AEs within 6 months of starting treatment, revealing that consolidation durvalumab was well tolerated following sCRT. In terms of survival, median PFS and OS were 10.9 and 25 months, respectively. In general, cCRT followed by ICIs consolidation remains the first choice; however, consolidation after sCRT is also a priority treatment option in frailer populations if cCRT cannot be tolerated.

The combination of monoclonal antibodies has demonstrated a sustained long-term response and survival in patients with stage IV disease [58–60]. In theory, if tolerable, a dual combination of immune strategies in the consolidation setting with complementary mechanisms of action may also overcome resistance to anti-PD-(L)-1 antibodies and further enhance the benefits of immunotherapy in LA-NSCLC. CTLA-4 suppresses T cell activity and inhibits immune responses by inhibiting the binding of the costimulatory molecules CD80 or CD86, found on the surface of antigen-presenting cells (APCs) in the tumor-draining lymph nodes (TDLNs), to the coactivation receptor CD28 [61]. It has been found that RT to a metastatic site of NSCLC could act as an in situ vaccine and synergize with anti-CTLA-4 antibodies [62]. Furthermore, anti-CTLA-4 inhibitors might mitigate the immunosuppressive effects exerted by irradiation on the TDLNs, thus of particular attention [63]. RT and dual checkpoint blockade, which is expected to cross the borders of PACIFIC, have gained attention. The open-label, randomized, phase II BTCRC-LUN 16–081 trial was designed to explore the combination of nivolumab plus ipilimumab for a shorter treatment duration of 6 months as consolidation treatment compared to nivolumab alone after cCRT [64]. A total of 115 patients were randomized to receive nivolumab (arm A) or nivolumab plus ipilimumab (arm B) after completion of CRT. The percentage of patients completing the full 6 months of treatment was 70.4% in arm A and 56.9% in arm B (*P* = 0.15). Despite a shortened interval of ICIs treatment, an improved 18-month PFS was achieved in both arms (63.7% in arm A and 67.6% in arm B) compared with historical controls (18-month PFS of 30%). The median PFS and 2-year OS rates were both similar in arm A and arm B. However, the incidence of grade 3 AEs (52.9% vs. 38.9%), grade 3 TRAEs (27.5% vs. 18.5%), and grade 3 pneumonitis (17.6% vs. 9.3%) were higher in arm B than in arm A. It appears that the combination shows no additive value in this setting. The phase III CheckMate 73 L trial will compare the PFS and OS of nivolumab plus cCRT, nivolumab plus ipilimumab consolidation (arm A), nivolumab plus cCRT, nivolumab consolidation (arm B), and the standard PACIFIC strategy (arm C) in unresectable stage III NSCLC [65].

Monalizumab is an immunoglobulin that targets NKG2A receptors. The COAST trial was a three-arm randomized (1:1:1) phase II study of consolidation durvalumab alone (control, arm C) or in combination with the anti-CD73 monoclonal antibody oleclumab (arm A) or the antiNKG2A monoclonal antibody monalizumab (arm B) in LA-NSCLC patients without disease progression after cCRT [66]. After a limited median follow-up of 11.5 months, ORR was 17.9%, 30.0% and 35.5% in the control arm, arm A, and arm B, respectively. PFS was significantly prolonged in both combinations compared to durvalumab alone. The incidence of serious TRAEs, all-cause grade ≥ 3 AEs, and all-grade pneumonitis were similar between the treatment arms. The clinical benefit of the combinations appeared to be persistent in an exploratory subgroup analysis, regardless of PD-L1 status, which certainly needs to be further verified in more patients. Indeed, the durvalumab arm in the COAST trial heavily underperformed as compared to that in the PACIFIC trial, which may be mainly related to different patient characteristics. In general, such combination approaches are feasible, safe, and may have the potential to improve the prognosis of patients with LA-NSCLC. Notably, the currently recruiting phase III PACIFIC-9 trial will further evaluate these combinations. The T-cell immunoglobulin and immunoreceptor tyrosine-based inhibition motif domain (TIGIT) is a novel inhibitory immune checkpoint expressed on CD8 + T cells and NK and T regulatory cells in multiple cancers [67]. Cancer cells and cancer antigen-presenting cells express CD155 and CD112, which bind TIGIT, decreasing T cell activity, and coordination with PD-1 or PD-L1 inhibitors shows promising early results [68]. Based on the improved ORR with atezolizumab plus the anti-TIGIT antibody tiragolumab, compared to atezolizumab and placebo, in the metastatic NSCLC setting, two randomized phase II clinical trials, the PACIFIC-8 with domvanalimab (AB154) plus durvalumab and the Skyscraper-03 with tiragolumab plus atezolizumab, are testing this approach as consolidation treatment after cCRT in unresectable stage III NSCLC. However, as the combination of atezolizumab plus tiragolumab has neither been reported to improve the PFS in the first-line setting in PD-L1 ⩾50% metastatic NSCLC (phase III RCT, SKYSCRAPER-01), PFS, and OS in advanced small cell lung cancer (phase III RCT, SKYSCRAPER-02), the anti-TIGIT enthusiasm has now decreased [69]. In our view, anti-TIGIT in stage III NSCLC is still unclear, as patients with stage III NSCLC represent a different patient population.

### Concurrent ICIs with CRT

Upon the success of ICIs consolidation following CRT, a series of upcoming clinical trials are investigating novel approaches to explore the best mode of application of ICIs in patients with LA-NSCLC. Representative completed and ongoing clinical trials investigating RT combined with ICIs are shown in Table 1.

**Table 1:** Representative phase II/III RCTs with iRT in unresectable LA-NSCLC

NCT identifier	Phase	Status	N	ICIs	Treatment arms	Primary endpoint	Outcomes		
							**PFS**	**OS**	**AEs (3–5)**
**cCRT or sCRT followed by ICIs consolidation treatment**									
NCT02125461 (PACIFIC) [9, 49, 70]	III	Completed	713	Durva	CCRT > durva vs. cCRT > placebo	PFS, OS	Median 16.9 vs. 5.6 5-yr 33.1% vs.19.0%	Median 47.5 vs. 29.1 5-yr 42.9% vs.33.4%	30.5% vs. 26.1%
NCT02343952 (LUN14-179) [52]	II	Completed	92	Pembro	CCRT > pembro	TMDD	Median 18.7	Median 35.8	4.3%
NCT03728556 (GEMSTONE-301) [55, 56]	III	Completed	381	Suge	CCRT/sCRT > suge vs. cCRT/sCRT > placebo	PFS	Median 10.5 vs. 6.2	Median NR vs. 25.9 3-yr 55.8% vs. 29.5%	9.0% vs. 6.0%
PACIFIC-6 (NCT03693300) [57]	II	Completed	120	Durva	SCRT > durva	Grade ≥ 3 AEs	Median 10.9 1-yr 49.6%	Median 25 1-year 84.1%	20.5%
NCT03706690 (PACIFIC-5)	III	Ongoing	400	Durva	CCRT/sCRT > durva vs. cCRT/sCRT > placebo	PFS	-	-	-
NCT03379441	II	Ongoing	126	Pembro	CCRT/sCRT > pembro	OS	-	-	-
NCT03728556	III	Ongoing	381	CS1001	CCRT/sCRT > CS1001 vs. cCRT/sCRT > placebo	PFS	-	-	-
NCT04325763	III	Ongoing	315	TQB2450	CCRT/sCRT > TQB2450 + anlotinib vs. CCRT/sCRT > TQB2450 vs. CCRT/sCRT > placebo	PFS	-	-	-
**BTCRC-LUN 16–081 (NCT03285321) **[64]	II	Completed	108	Nivo/ipili	CCRT > nivo + ipili vs. cCRT > nivo	PFS	Median 25.4 vs. 25.8	Median NR 2-yr 78% vs. 81%	52.9% vs. 38.9%
**COAST (NCT03822351) **[66]	II	Completed	189	Durva/olec/mona	CCRT > durva + olec vs. cCRT > durva + mona vs. cCRT > durva	ORR	Median NR vs. 15.1 vs. 6.3 1-yr 62.6% vs. 72.7% vs. 33.9%	Median NR	40.7% vs. 27.9% vs. 39.4%
**NCT04513925** **(Skyscraper-03)**	III	Ongoing	800	Atezo/tirago/durva	CCRT > atezo + tirago vs.cCRT > durva	PFS	-	-	-
**NCT04026412** **(Checkmate 73L) **[65]	III	Ongoing	888	Nivo/ipili/durva	CCRT + nivo > nivo + ipili vs. cCRT + nivo > nivo vs. cCRT > durva	PFS, OS	-	-	-
**NCT05221840 (Checkmate-9)**	III	Ongoing	999	Durva/olec/ mona	CCRT > durva + olec vs. cCRT > durva + mona vs. cCRT > durva	PFS	-	-	-
**NCT04905316 (CHORUS)**	II	Ongoing	32	Durva/ canaki	CCRT + canaki > canaki + durva	PFS	-	-	-
**Combination of cCRT and ICIs followed by ICIs maintenance treatment**									
NCT03631784 (KEYNOTE-799) [71, 72]	II	Completed	216	Pembro	CCRT + pembro > pembro	ORR	Arm A Median 30.6 1-yr 67.1% 2-yr 55.3%	Arm A Median NR 1-yr 81.3% 2-yr 64.3%	Arm A 64.3% Grade ≥ 3 pneumonitis 8.0%
							Arm B NR 1-yr 76.6% 2-yr 60.6%	Arm B Median NR 2-yr 87% 2-yr 71.2%	Arm B 50.0% Grade ≥ 3 pneumonitis 6.9%
NICOLAS (NCT02434081) [73, 74]	II	Completed	79	Nivo	CCRT + nivo > nivo	Grade ≥ 3 pneumonitis	1-yr 53.7% Median 12.7	1-yr NR Median 38.8	Grade ≥ 3 pneumonitis 11.7%
NCT02525757 (DETERRED) [75]	II	Completed	52	Atezo	CCRT + atezo (sequential) > atezo vs. CCRT + atezo (concurrent) > atezo	Grade ≥ 3 AEs	Median 18.6 vs. 13.2 1-yr ~ 55% vs. ~ 52%	Median 22.8 vs. NR 1-yr ~ 80% vs. ~ 80%	80% vs. 80%
NCT03519971 (PACIFIC 2)	III	Ongoing	328	Durva	CCRT + durva > durva vs. cCRT + placebo > placebo	PFS	-	-	-
NCT03945227 (PASTURE)	II	Ongoing	200	PDR001	CRT + PDR001 > PDR001 vs. CRT > PDR001	PFS	-	-	-
NCT04092283	III	Ongoing	660	Durva	CCRT + durva > durva vs. cCRT + placebo > durva	OS	-	-	-
NCT04380636 (KEYLYNK-012)	III	Ongoing	870	Pembro/olaparib/durva	CCRT + pembro > pembro vs. cCRT + pembro > pembro + olaparib vs. cCRT + durva	PFS, OS	-	-	-
NCT04085250	II	Ongoing	264	Nivo	CT + nivo > cCRT + nivo > nivo vs. CT + nivo > cCRT + nivo > FU	PFS	-	-	-
NCT03840902	III	Ongoing	350	Durva/ M7824	CCRT + M7824 > M7824 vs cCRT + durva	PFS	-	-	-
NCT04765709 (BRIDGE trial)	II	Ongoing	65	Durva	CT + durva > durva + RT > durva	PFS	-	-	-
**CCRT following induction ICIs followed by ICIs maintenance treatment**									
NCT03102242 (AFT-16) [76]	II	Completed	64	Atezo	Atezo > CRT > atezo	DCR	Median 23.7 1-yr 66%, 18 mo 57%	Median NR 18 mo 84%	Median NR
NCT03523702 (SPRINT) [77]	II	Ongoing	63	Pembro/ durva	Pembro > RT > pembro (PD-L1 ≥ 50%) vs. cCRT > durva (PD-L1 < 50%)	PFS	PD-L1 ≥ 50% 1-yr 73%	PD-L1 ≥ 50% 1-yr 91%	Median NR
NCT04776447 (APOLO)	II	Ongoing	51	Atezo	Atezo + CT > cCRT > atezo	PFS	-	-	-
NCT04364048	II	Ongoing	54	Durva	Durva > cCRT > durva	PFS	-	-	-
NCT04230408 (PACIFIC BRAZIL)	II	Ongoing	48	Durva	Durva + CT > cCRT + durva > durva	PFS	-	-	-
NCT05128630 (DEDALUS)	II	Ongoing	45	Durva	CT + durva > RT + durva > durva	Safety	-	-	-

Nearly half of the patients with unresectable stage III NSCLC who received CRT did not meet the PACIFIC criteria for durvalumab eligibility [78, 79]. The most common reason for durvalumab ineligibility was disease progression during CRT followed by therapy-related pneumonitis [78]. Concurrent ICIs with CRT would offer all patients eligible for cCRT the opportunity to receive ICIs and may also exploit the potential synergism between chemotherapy and ICIs [80, 81]. In a phase I trial to study the safety and efficacy of using pembrolizumab concurrently with CRT in LA-NSCLC, the 12-month PFS rate was 69.7%, higher than the 55.7% reported in the PACIFIC trial, and the median OS and 1-year OS rate were 29.4 months and 85.2%, respectively [82]. The incidence of irAEs above grade 2 was 67% and the incidence of pneumonia above grade 2 was 33%. The phase II, nonrandomized, 2-cohort (arm A, squamous and non-squamous histology; arm B, non-squamous histology), open-label KEYNOTE-799 study showed that pembrolizumab plus cCRT provided robust anti-tumor activity (ORR, 70.5%) with a manageable safety profile for patients with previously untreated, stage III, unresectable LA-NSCLC, regardless of tumor histologic type and PD-L1 expression [71]. In the most recent two-year update, a median PFS of 30.6 months was reported in cohort A with 2-year OS of 64.3%, and the median PFS was not reached in cohort B, with 2-year OS of 71.2% [72]. DETERRED is a phase II study designed to evaluate the efficacy and safety of atezolizumab combined with cCRT in patients with LA-NSCLC [75]. Part 1, with 10 evaluable patients, was administered conventionally fractionated cCRT followed by two cycles of chemotherapy plus atezolizumab, followed by consolidation atezolizumab for up to 1 year. Part 2, with 30 evaluable patients, involved the administration of cCRT concurrently with atezolizumab followed by the same maintenance therapies as in part 1. The median OS in part 2 has not yet been reached, suggesting better efficacy than combining atezolizumab sequentially with cCRT in part 1. The single-arm phase II NICOLAS trial evaluated the use of nivolumab concomitant with cCRT in 79 LA-NSCLC patients [73]. After receiving one cycle of chemotherapy, the patients were treated with two cycles of nivolumab concurrently with cCRT, and then treated with nivolumab as consolidation therapy for 12 months. Overall, nine (11.7%) patients experienced grade 3 pneumonitis. The 1-year PFS was 53.7% (95% CI, 42.0–64.0) with a median PFS of 12.7 months (95% CI, 10.1–22.8), and the median OS was 38.8 months (95% CI, 26.8–NR) [74]. The results of the three non-randomized phase II trials cannot be compared directly with PACIFIC (randomization after cCRT) due to the design and small sample sizes. However, these data suggest the feasibility and safety of the concurrent administration of ICIs and cCRT in LA-NSCLC. Except for KEYNOTE-799, the 1-year PFS was approximately equal to that of PACIFIC. Of course, the actual efficacy of the triplet regimen should be compared with the PACIFIC status in phase III randomized controlled trials.

Further insight is expected from the ongoing phase III KEYNOTE-012 study comparing pembrolizumab plus cCRT followed by pembrolizumab with or without the poly adenosine diphosphate-ribose polymerase (PARP) inhibitor olaparib with cCRT followed by durvalumab in patients with unresectable stage III LA-NSCLC. KEYVIBE-006 evaluated MK-7684A (co-formulation of vibostolimab-anti-TIGIT plus pembrolizumab) plus cCRT, followed by MK-7684 versus cCRT followed by durvalumab. Other phase III clinical trials, including the CheckMate 73 L (concurrent nivolumab with or without ipilimumab followed by nivolumab), ECOG-ACRIN EA5181 (concurrent and consolidation durvalumab), and PACIFIC2 trial (concurrent and consolidation durvalumab) are also ongoing. The results of these trials will elucidate whether more intensive treatment improves outcomes without compromising safety.

### Induction ICIs followed by CRT

Similarly, induction ICIs followed by CRT also enable more patients to benefit from ICIs treatment. Moreover, both RT and chemotherapy play a double-edged role in the immune system. Effective T cell infiltration triggered by RT or chemotherapy only occurs when the immune system is not destroyed. The direct killing effect of RT and chemotherapy on circulating lymphocytes and stem cells cannot be disregarded [5, 83]. Hence, the approach of using ICIs before CRT has the advantage of an intact and healthy immune system. The AFT-16 trial is the first phase II trial to explore induction ICIs before CRT in stage III NSCLC [76]. Enrolled patients first received two cycles of atezolizumab and were then restaged. Two more atezolizumab treatments were delivered if not progressive, followed by standard cCRT and consolidation atezolizumab for up to 1 year. Patients who had progressed at the first restaging point immediately received cCRT. The primary endpoint was the disease control rate at the end of induction atezolizumab, and an inspiring result of 77.4% was reported. A remarkable median PFS of 23.7 months was observed. The PFS at 12 months after the completion of cCRT was 78%, which is impressive compared with PACIFIC (12 months PFS = 55%). Although the AFT-16 population was highly selected, the study did not limit the eligibility to responders to cCRT, as in the PACIFIC trial [9, 49, 70]. Similarly, the ongoing phase II SPRINT trial is evaluating a chemotherapy-free strategy in PD-L1 ⩾50% tumors (*n* = 25), with sequential three cycles of induction pembrolizumab followed by risk-adapted thoracic RT and followed by 12 additional cycles of pembrolizumab [77]. The trial also enrolled patients with tumors with PD-L1 expression < 50% who were treated with standard cCRT to serve as a non-randomized comparison group (*n* = 38). In the first interim analysis of patients with PD-L1 ⩾50%, 48% achieved partial response (PR), with 1-year PFS and OS rates of 73% and 91%, respectively. Intriguingly, after three cycles of pembrolizumab induction, patients with PR at the restaging positron emission tomography/computed tomography (PET-CT) (*n* = 12) had a 1-year PFS of 100%, compared to 61% in patients with stable or progressive disease. Thus, the response observed by PET following pembrolizumab induction may be useful for identifying patients who can be successfully treated without chemotherapy. Similarly, the NRG-LU004 trial assesses the combination of durvalumab concomitantly with RT followed by durvalumab for 1 year in patients with PD-L1 ⩾50% NSCLC. The phase II APOLO trial assessed neoadjuvant atezolizumab plus chemotherapy followed by cCRT and the maintenance of atezolizumab for 12 months. Another phase II trial evaluated the same treatment strategy as nivolumab. The difference is that a comparator arm without nivolumab maintenance was included. Moreover, three single-arm phase II trials (NCT05128630, NCT04765709, and PACIFIC-BRAZIL) evaluated the induction of durvalumab plus chemotherapy, followed by RT (cCRT in PACIFIC-BRAZIL) concurrently with durvalumab followed by durvalumab consolidation. We are conducting a phase II, multicenter, randomized, open-label, controlled trial comparing induction treatment with camrelizumab combined with chemotherapy following cCRT and maintenance camrelizumab with standard CRT for LA-NSCLC. It is expected that ICIs and chemotherapy can work synergistically to better play the role of neoadjuvant therapy. In addition, it has been proven in resectable NSCLC that ICIs combined with chemotherapy can achieve tumor downstaging and provide a more supportive immune microenvironment [84]. Thus, a reasonable hypothesis would be that induction ICIs and chemotherapy in unresectable LA-NSCLC can not only downsize initial tumors, but also reduce resistance to RT. The preliminary results demonstrated that induction ICIs with chemotherapy followed by radical cCRT yielded an inspiring median PFS of 20.4 months for unresectable LA-NSCLC, which was markedly superior to most of the results in the aforementioned studies with other treatment regimens.

### Toxicity and safety

Enhanced anti-tumor immune surveillance upon treatment with ICIs is inherently at the expense of immune-related adverse events (irAEs), which affect virtually every organ system and are a proposed Achilles’ heel of this class of therapeutic agents [85, 86]. It is estimated that 50% of patients treated with ICIs will experience some form of irAEs [85, 87]. Compared to other irAEs, ICIs-related pneumonitis (CIP) is characterized by rapid onset and high fatality, warranting early detection. CIP is defined as the development of dyspnea and/or other respiratory symptoms in the presence of new infiltrates on chest imaging without the presence of new infections[88]. Chest CT scans currently play a major role in the diagnosis of CIP because of the difficulty in predicting the development of irAEs prior to starting therapy [89]. A meta-analysis reported that the incidence of all-grade CIP during PD-1 inhibitor monotherapy for NSCLC was 4.1%, which is higher than the overall incidence of multiple advanced cancers [90]. Similarly, the reported incidence of any grade pneumonitis without RT was 3.8% in the largest pooled analysis to date of AEs risk associated with the use of RT prior to ICIs [91]. Moreover, patients who received RT before ICIs had similar rates of various AEs, including pneumonitis. However, the incidence of CIP was found in 10% of the entire population from a single institute retrospective study, higher than that reported for other irAEs (1–5% of all malignancies) [88]. The population included 151 lung cancer patients and the incidence of CIP was even higher (12.6%) in these patients. This discrepancy could mainly arise from the drawbacks of the small sample. On the other hand, a higher incidence in lung cancer patients could arise from the dominance of male lung cancer patients, a large proportion of whom are smokers [92]. Additionally, combination immunotherapy (vs. monotherapy), the use of PD-1 inhibitors (vs. PD- L1 inhibitors), and the use of ICI as a first-line therapy (vs. second-line or further) are also associated with a higher risk of CIP [93]. Notably, the incidence of other irAEs was also significantly higher in patients with patients who did not develop CIP [88]. In general, due to the variety of local or systemic treatments that act together on lung tissue, CIP in NSCLC is worthy of close attention.

A phase II study with multisite SBRT and pembrolizumab treatment, as well as the PEMBRO-RT phase 2 randomized trial, showed concordant results with tolerable irAEs [10, 94]. As reported in the PACIFIC trial, grade 3 or 4 AEs occurred in 29.9% of patients who received durvalumab after cCRT; the most common grade 3 or 4 AE was pneumonia, with an incidence of 4.4% [9]. A total of 15.4% of the patients discontinued durvalumab because of AEs. In the GEMSTONE-301 trial, grade 3 or 4 treatment-related irAEs occurred in 22 (9%) of 255 patients in the sugemalimab group compared with seven (6%) of 126 patients in the placebo group [55]. The most common, pneumonitis or immune-mediated pneumonitis, occurred in 7 (3%) patients. The rate of grade 2 or higher pneumonitis was 10% in the DETERRED trial, demonstrating good tolerance [75]. However, a secondary analysis by KEYNOTE 001 proposed that the treatment-induced pulmonary toxicity rate differed between the two groups (13% with combination vs. 1% with ICIs only, *P* = 0.046) [95]. Another multicenter analysis of safety and toxicity reported that the rates of related subacute grade ≥ 3 irAEs in the SBRT combined with ICIs and SBRT alone groups were 26.8% and 2.9%, respectively, and the rates of grade ≥ 3 pneumonitis were 10.7% vs. 0 with *P* < 0.01 [96]. IrAEs can also induce fatal outcomes, reminding us of cautious surveillance. Many factors could contribute to the disparate results, such as prior lung disease, prior treatment, previous or current smoking, age > 70 years, type of inhibitor, and histological type [97]. Moreover, the timing of RT may be important, and the sequence of combining RT and ICIs remains controversial.

Interestingly, several publications have proposed that irAEs might be related to significantly better ORR, PFS, and OS in NSCLC patients who received PD-1/PD-L1 inhibitor monotherapy [87, 98, 99]. In combination therapy, this finding was also observed. In a retrospective study of 201 patients with nivolumab combined with prior thoracic RT, longer mPFS and lower disease progression rates were found in those who experienced therapy-associated pneumonitis compared with those who didn’t (3.6 vs. 2.3 months, *P* = 0.023; 29.4% vs. 47.9%, *P* = 0.059) [100]. Hwang et al. proposed that patients with grade 2 or higher irAEs, especially pneumonitis, had better survival benefits [101]. Based on preclinical theory, some studies have speculated that the occurrence of irAEs might reflect a much more active immune response, indicating a strong anti-tumor immunity function under combination treatment [89]. Therefore, the observation of irAEs may not only be induced by overlapping toxicity, but also contribute to outcome prediction.

### Progression during and after ICIs consolidation therapy

Resistance to ICIs remains a key clinical barrier to further improving the outcomes of patients with advanced or metastatic lung cancer. Approximately 80% of patients with unselected advanced NSCLC do not respond to single-agent nivolumab [102]. As mentioned above, progression after ICIs discontinuation is classified into a distinct resistance scenario, from primary or secondary resistance [41]. Progression during ICIs therapy can be defined based on the presence of more than 6 months of disease control. In terms of LA-NSCLC, more than half of the patients would progress within 2 years of the start of treatment [49, 55]. Updated data from the PACIFIC trial showed that 49.0% of patients completed 12 months of ICIs treatment, and 31.3% discontinued owing to disease progression [49]. In the PACIFIC trial, 7.1% of patients in the ICIs arm received durvalumab retreatment and completed the initial 12 months of durvalumab with disease control and progressed during follow-up, and the median time to second progression measured from the random assignment was 48.0 months [49]. Subsequent ICIs were less commonly used among patients randomly assigned to the durvalumab arm than those in the placebo arm (12.6% vs. 29.1%). A real-world multicenter retrospective study of 116 patients with unresectable stage III NSCLC treated with CRT followed by at least one dose of durvalumab reported no significant difference in response and time of treatment with combined chemotherapy and ICIs vs. chemotherapy alone, which was posted in 2022 European Lung Cancer Congress [103]. Another phase II, single-arm, multi-center trial of consolidation pembrolizumab for up to one year following concurrent chemo-RT in unresectable stage III NSCLC reported in the 2019 World Lung Cancer Congress that response rates with chemotherapy were similar to what is expected in the second-line setting for patients with disease progression after consolidation pembrolizumab, and only 1 of 6 patients rechallenged with ICIs responded [104]. Apart from these, no other data were available on rechallenge with ICIs at progression after completion of 12 months of durvalumab treatment in LA-NSCLC. Therefore, rechallenge with ICIs or immunochemotherapy was less encouraging at progression during and after the ICIs consolidation therapy in a locally advanced setting.

The relative prevalence of oligometastatic disease is estimated to range from 30 to 50% in advanced NSCLC [105]. Oligoprogression, a more specific concept, is increasingly encountered in patients treated with ICIs, which may be a common pattern of acquired resistance to ICIs [106, 107]. Unlike systemic treatment options, RT not only eradicates local lesions but also plays a role in overcoming resistance to ICIs. In a retrospective study of 26 patients with acquired resistance to anti-PD-1 inhibitors, 88% had recurrence limited to one (54%) or two (35%) sites, and local RT to oligo-progression with the continuation of ICIs achieved superior survival [108]. Similarly, local RT plus continued ICIs led to significantly longer PFS and OS in patients with oligo-progression from ICIs treatment compared with those who received no local RT [109]. In theory, chemo-RT provides a supportive TME for ICIs consolidation treatment, reducing primary or acquired resistance to ICIs to some extent, while the addition of RT during or after ICIs helps overcome the developed resistance. Promising clinical evidence highlights the superiority of iRT in LA-NSCLC and the role of RT in oligo-progressive NSCLC after ICIs treatment.

### Challenges, strategies, and auspicious orientations

#### Optimal dose and fractionation of RT

Regarding RT, the dose and fraction scheme are essential in the lesion local control and outcome, while enhanced immunity probably plays a mediating role. Hyperfractionation RT (HyperRT) and hypofractionated RT (HypoRT) are the concepts of conventional fractionation. SBRT is a typical representation of hypo-RT, consisting of the administration of high doses of RT with a narrow margin and a strong gradient to protect the surrounding healthy tissues, also known as stereotactic ablative radiotherapy (SABR). Siva et al. proposed that conventional fractionation was detrimental to RT-induced anti-tumor immune responses, as irradiation intervention would frequently purge local immune lymphocytes [110]. Their research demonstrated that a single high-dose RT could release more TAAs without depleting immunocytes, shielding CD8 + T cells and NK cells to a certain extent [110]. Chen et al. also confirmed that SBRT can better protect lymphocytes than conventional fractionation [111]. However, a higher dose in a single fraction is not always preferable. However, an excessive dose in a single fraction may induce a suppressive TME. It has been reported that high dose irradiation such as 20 Gy induced expression of three prime repair exonuclease 1 (Trex1), which bears the function of degrading the dsDNA in the cytoplasm, and thereby weakened the immunomodulatory effects of RT [112]. Therefore, SBRT has been the standard therapy for early NSCLC patients who are not suitable for surgery, allowing the delivery of high doses to relatively small target lesions [8, 113]. Notably, the latest revised STARS study provided a higher level of evidence for its use in patients with operable early NSCLC [114]. In the randomized phase I/II MDACC trial for metastatic NSCLC with lung and liver lesions, compared to traditional RT with 45 Gy in 15 fractions, 50 Gy in 4 fractions led to better out-of-field ORRs (38% vs. 10%) and longer median PFS (20.8 vs. 6.8 months) when combined concurrently with pembrolizumab [115]. The pooled analysis of the PEMBRO-RT (24 Gy in 3 fractions, sequential with pembrolizumab) and MDACC trials showed that pembrolizumab plus RT with 50 Gy in 4 fractions corresponded to the best PFS [17]. However, better survival over 24 Gy in three fractions may mostly derive from the concurrent delivery of RT and ICIs. In conclusion, these data indicate that SBRT and HypoRT are not only prominent in local control but can also better coordinate the effect of ICIs. In addition, it seems that a high single dose of 8–10 Gy is the optimal dose to activate the anti-tumor immune response, in contrast to conventional fractionation [5, 37]. This hypothesis warrants further corroboration in a dedicated, large-volume, phase III, randomized trial.

The current standard of CRT for unresectable LA-NSCLC consists of 6–7 weeks of RT with a dose of 60–70 Gy in 2 Gy daily fractions and chemotherapy administered at a reduced dose, as opposed to the systemic dose when chemotherapy is administered by itself [8]. The RTOG 0617 trial reported the highest OS (5-year OS rate, 32.1%) of any phase III trial without the addition of ICIs for stage III NSCLC patients, strongly supporting a SoC RT with 60 Gy given to a target volume directed at the tumor plus margin on the basis of CT and PET/CT, excluding elective nodal irradiation (ENI) [116]. Secondary analysis suggested that a higher RT dose to immune cells correlated with worse tumor control and OS [117]. However, the controversy regarding the benefits of dose escalation remains open. These poor results may be attributed to the prolongation of the global treatment time, which leads to an accelerated repopulation of cells. A meta-analysis examining different RT schemes, including regimens with splits, hypoRT, hyperRT, and dose escalation with conventional fractionation, found that an increased biologically effective dose administered without chemotherapy improved survival [118]. Therefore, the role of SBRT in LA-NSCLC has become an area of great interest. Several studies have examined a combination of conventional and SBRT boost for locally advanced disease, but there are limited data regarding SBRT as a complete replacement for conventional radiation. Recently, safety results of NRG-LU004 reported that chemotherapy-free thoracic accelerated fractionated RT (60 Gy/15F) was safe when administered with concurrent durvalumab in LA-NSCLC patients with high PD-L1 expression [119]. Another single-arm phase II study showed that a combination of SBRT and systemic dose chemotherapy was a safe and effective treatment for LA-NSCLC [120]. Of course, the results warrant further investigation, owing to the small sample size.

SBRT may increase local control in patients with LA-NSCLC with an acceptable safety profile, although the level of evidence is still deficient. Further, given the immunomodulatory role of RT, especially SBRT, it is presumable that novel treatment schemes for LA-NSCLC integrating hypoRT or SBRT with ICIs will be proposed in the coming years. Moreover, low-dose irradiation (LDI) ranging from 0.5 Gy to 2 Gy has recently been proposed, which can reshape the TME, including the polarization of M1 macrophages and homing of T cells. The results of a clinical trial of SBRT combined with ipilizumab for advanced malignant tumors found that tumors exposed to low-dose scattered radiation (close to the SBRT-targeted region) were more likely to respond to therapy than lesions far from the target [121]. Based on these, a novel treatment modality with LDI combined with SBRT was proposed, in which SBRT irradiates primary lesions to ignite the “in situ vaccine” effect and LDI reshapes the stroma of other metastases [122]. Recently, a novel strategy of high- and low-dose RT combined with anti-TIGIT and anti-PD1 monoclonal antibodies in lung adenocarcinoma cell lines revealed further improved efficacy, providing a new treatment alternative for cases refractory to other checkpoints [123]. Hence, a high-plus low-dose RT strategy for LA-NSCLC may also be worth exploring. Notably, although controversial, single-fraction SBRT has also been evaluated for negative regulation of TME in peripheral early-stage NSCLC and metastatic lesions [124, 125]. Of course, there is a long way to go for the use of SBRT or even one-stop SBRT in LA-NSCLC.

### RT target and target volume

It is certain that a consensus on the definition of the target volume is key to avoiding excess toxicity due to large volumes. As mentioned before, the RTOG 0617 trial excluded ENI without targeting areas of high FDG uptake and denied dose escalation [116]. TDLNs are important sites for the activation and accumulation of anti-tumor T lymphocytes; therefore, ENI may affect the adaptive immune response to some extent. Accordingly, by irradiating TDLNs, the adaptive immune response was attenuated in a transplantable mouse model treated with SBRT and ENI, especially when RT and ICIs were combined [126]. A multicenter open-label, randomized, controlled trial PET-Plan (ARO-2009–09) suggested that [18] F-FDG PET-based planning could potentially improve local control without increasing CRT-related toxicity in patients with LA-NSCLC. Our phase II randomized trial also found that [18] F-FDG PET/CT adaptive shrinking field and simultaneous integrated boost RT technique can improve ORR, OS, and PFS without increasing the risk of RT-related toxicity [127]. However, van Diessen et al. reported higher rates of acute and late toxicity in a randomized phase II dose escalation trial that used PET boost. The results of the RTOG 1106 trial were also reported, in which a mid-treatment PET/CT was used to allow a hypofractionated boost over the last 2 weeks to escalate the RT dose to residual disease, failing to achieve improved local control and OS [128]. In general, randomized data support the omission of ENI from PET information, but whether to boost the RT dose deserves further investigation.

### Charged particle therapy

Charged particle therapies, such as protons and heavy ions, have rapidly developed to play a vital role in tumor therapy. Compared with photon RT, the most notable feature of charged-particle therapy is the sharper dose distribution derived from the spread-out Bragg peak, which could significantly reduce the radiation beam on adjoint normal tissues. Both early stage and LA-NSCLC are suitable for proton RT [129]. However, there is a lack of strong evidence to prove the superiority of proton RT over photon RT. Some phase I/II trials are attempting to hypofractionate RT dose by proton RT, with a focus on the ability of protons to limit the normal tissue dose [130]. Another ongoing phase III trial, RTOG 1308, comparing photons to protons, allows a higher dose of 70 Gy to be delivered to the appropriate arm when normal tissue dose constraints are met. Carbon-ion RT (CIRT), a type of heavy ion RT, not only has the Bragg peak character but also influences the immune response differently from photon RT. Preclinical studies have proposed that carbon-ion beams render complex and difficult-to-repair DNA double-strand breaks in irradiated tumor cells [131]. This could enhance the release of HMGB1 by increasing linear energy transfer in tumor cells, indicating that the combination of ICIs and CIRT is a promising point to investigate.

### Choice, timing, and duration of ICIs

Although numerous trials have confirmed that combined ICIs and CRT would benefit patients with LA-NSCLC, the optimum mode of combination therapy remains controversial. The first question was which type of ICI could match better with CRT. In metastatic NSCLC, two single-institution prospective trials showed a significantly better PFS with anti-PD-1 combined with SBRT than with anti-CTLA4 (6-month PFS, 87% vs. 52%; 18-month PFS, 63% vs. 23%; *P* = 0.02) [132]. In terms of LA-NSCLC, all published and ongoing trials have selected anti-PD-(L)-1 antibodies as monotherapy for efficacy intensification. Interestingly, the optimal timing of anti-CTLA4 combined with RT was different from that of anti-PD-(L)-1 antibodies. The stimulatory effect of RT on the TME can be exploited when anti-PD-(L)-1 antibodies are used concurrently with or after RT because they function to limit T cell activity, whereas anti-CTLA4 targeting Tregs should be administered before RT to assist antigen presentation [133, 134]. For patients with LA-NSCLC, ICIs may be administered before, after, or concurrently with RT. PACIFIC has laid a framework for administering ICIs after RT in this setting [9]. Subgroup analysis of the PACIFIC study showed that receiving ICIs within 0–14 days after the end of cCRT correlated with better PFS and OS than patients receiving ICIs between 15–42 days [9]. Similarly, a retrospective observational study of patients with stage III unresectable NSCLC who received durvalumab after cCRT from 2018 to 2021 was recently reported; patients who received durvalumab 30–60 days after cCRT had lower OS rate at 30 months compared to those who started durvalumab before 30 days (44% vs. 90%) [135]. However, this difference was not statistically significant (*P* = 0.45). Additionally, as the TROG1937 (DATE study, jRCTs031190117) reported, a phase II study, durvalumab can be safely administered immediately after completion of cCRT for patients with unresectable stage III NSCLC, with no additional or unexpected toxicity as a reference to PACIFIC [136]. In contrast, a retrospective analysis of 371 patients treated with ICIs after SBRT showed that administration of ICIs for at least 21 days after SBRT had longer OS [133]. However, this was a retrospective study with several confounding factors. In contrast, the pooled analysis mentioned before of AEs associated with the use of RT prior to ICIs demonstrated that patients receiving RT prior to ICI generally had similar rates of AEs compared with those who did not receive prior RT [91]. The administration of ICI within 90 days generated a slightly numerically higher rate of AEs, and this difference was attributed to low-grade AEs. Thus, they concluded that it would appear safe to administer ICI within 90 days of receiving RT. It appears that the administration of ICIs after RT is generally safe for both locally and advanced NSCLC patients. Nevertheless, a retrospective analysis of patients with prior irAEs found that thoracic RT resulted in a very high risk of clinically significant and persistent RP [137]. However, it remains unclear whether pneumonitis is caused by RT. With regard to ICIs concurrent with CRT, data from three main non-randomized phase II trials suggested tolerable toxicity and at least comparable efficacy in LA-NSCLC [71–75].

Taken together, both preclinical and clinical evidence tends to support RT prior to or concurrently with anti-PD-(L)-1 antibodies, and in consolidation schemes, it seems that early addition after RT improves survival. However, as elaborated previously, induction ICIs before RT also have unique advantages, such as the potential to mitigate resistance to RT and retain intact immunity. Concerns regarding toxic effects have focused on the synchronous administration of ICIs and CRT. There may be no difference in toxicities between the two sequential schemes before or after CRT. Which strategy is superior or inferior warrants a detailed comparison in prospective head-to-head trials. A comprehensive evaluation and consideration of the efficacy, side effects, and actual conditions of patients should be adopted in clinical practice. Specifically, when combined with anti-CTLA4, the mainstream view supports the delivery of ICIs prior to RT. Strategies with dual checkpoint blockade could consider using anti-CTLA4 first, which needs to be validated in randomized clinical trials.

The optimal treatment duration for ICIs consolidation remains to be determined. In the PACIFIC trial, durvalumab was scheduled to be administered every 2 weeks for up to 12 months; however, only 43% of the enrolled patients completed the planned therapy [9]. In the first-line advanced setting, PD-1 or PD-L1 inhibitor treatment often lasted up to 2 years (KEYNOTE-024, KEYNOTE-189) or until disease progression (IMpower150) [55]. Accordingly, in the GEMSTONE-301 trial, the patient received sugemalimab treatment for up to 24 months [55]. Nevertheless, the percentage of patients completing the 2 years of therapy is still unknown, as at the data cutoff, 43% of patients in the sugemalimab arm were still on treatment. We need to know whether a longer treatment duration correlates with a higher benefit from ICIs or whether a shorter duration of treatment is also feasible. A retrospective study of 1006 patients with stage III NSCLC who received cCRT and at least one dose of adjuvant durvalumab suggested that PFS was similar for 9 months versus 12 months of durvalumab treatment, and PFS for 6 months was inferior versus 12 months [138]. The most common reasons for early discontinuation were tumor progression (22%), irAEs (15%), and non-immune-related toxicities (6.0%). In the absence of conclusive evidence from prospective randomized controlled studies, further consideration is required to make clinical decisions. For example, opinions were divided on whether ICIs consolidation should be used in PD-L1 negative patients. Therefore, we may choose to administer a shortened course of ICIs treatment, especially when toxicities are severe. The role of the dynamic circulating tumor DNA (ctDNA), other than PD-L1 expression, is also of relevance. Growing evidence has demonstrated that ctDNA minimal residual disease (MRD) following treatment for solid tumors can predict relapse [139]. According to the MRD status after CRT, early intervention may be feasible in patients at high risk of progression, and the dynamic evolution of ctDNA carrying more information may facilitate personalization of the duration of ICIs. For NSCLC, a study of patients with metastatic NSCLC receiving pembrolizumab or a combination of pembrolizumab and chemotherapy indicated that serial monitoring of ctDNA may serve as a non-invasive predictor of response [140]. In LA-NSCLC, ctDNA has also been shown to predict significantly better clinical outcomes, which may allow for personalized adjuvant treatment [141]. In terms of RT, Bi et al. performed targeted next-generation sequencing (NGS) of serial plasma samples from NSCLC patients who received front-line CRT or RT and found that ctDNA collected 1 month after treatment was optimal for predicting patient survival [142]. As mentioned before, the BTCRC LUN 16–081 trial investigated a shortened interval (6 months) of treatment with nivolumab or nivolumab plus ipilimumab, and improved 18-month PFS was achieved in both arms [64]. Analysis of ctDNA in the BTCRC LUN 16–081 trial found that MRD-positive patients after completion of CRT were strongly associated with inferior PFS compared to MRD patients (1-year 29% vs. 76%, 2-year 29% vs. 68%, respectively, *P* = 0.003) [143]. Specifically, patients with undetectable MRD at the end of consolidation ICIs therapy demonstrated a 2-year OS of 91%. However, all patients with increasing ctDNA levels after two cycles of ICIs treatment experienced disease progression within 10.8 months of starting ICIs treatment. Similarly, a study applied ctDNA analysis to 218 samples from 65 patients with LA-NSCLC receiving CRT, and 28 patients receiving consolidation ICIs were included [144]. The results revealed that patients with undetectable ctDNA after CRT (no MRD) had excellent outcomes, regardless of whether they received consolidation ICIs. In contrast, patients with detectable ctDNA showed significant benefits with ICIs consolidation ICIs treatment. All these data suggest that MRD detection after CRT might be capable of distinguishing the cured population from the population who require enhanced ICIs consolidation. In addition, changes in ctDNA levels during consolidation treatment could serve as an early biomarker of disease progression and long-term outcomes. In an ongoing clinical trial (NCT04585490), consolidation ICIs will be personalized according to MRD after CRT. MRD-positive patients will receive four cycles of platinum doublet chemotherapy and durvalumab, whereas MRD patients will only receive durvalumab monotherapy.

### Oncogenic addicted tumors

To date, there is no current SoC for patients with oncogenic driven stage III NSCLC. In the stage IV setting, ICIs have not achieved the desired effect in the treatment of oncogene-driven NSCLC, such as *EGFR* exon 19 and 21 mutations and *ALK* and *ROS-1* rearrangements, either first- or second-setting [145, 146]. Although the data are derived mostly from subgroup analyses of prospective trials and retrospective studies, the mainstream does not support the use of ICIs in unresectable stage III NSCLC [147]. The PACIFIC trial enrolled 43 *EGFR*-mutant patients, the results of which were not promising, with a PFS HR of 0.84 (95% CI: 0.40–1.75) and OS HR of 0.97 (95% CI: 0.40–2.33) [9]. Recently, a post hoc exploratory analysis of the PACIFIC trial evaluated the efficacy and safety of durvalumab in 35 *EGFR*-mutant patients [148]. In this subgroup, neither the PFS nor the OS was observed to improve with durvalumab, compared with the placebo. The safety profile of durvalumab was consistent with that of the overall population. The incidence of RP was 42% in the durvalumab arm versus 36% in the placebo arm, and that of pneumonitis was 17% and 18%, respectively. A multicenter retrospective study enrolled 323 patients treated with CRT and consolidation durvalumab, 43 (23%) of whom had oncogenic driver alterations, mainly *KRAS* (*n* = 26), followed by *EGFR* (*n* = 8), *BRAF* (*n* = 5), and *ALK* (*n* = 4) [149]. They observed limited activity in patients with *EGFR* mutations (mPFS, 8.1 months) and *BRAF* V600E mutation/*ALK* rearrangements (mPFS, 7.8 months). Only those *KRAS*-mutant tumors (*n* = 26) benefited from durvalumab maintenance. Another multi-institutional retrospective analysis (*n* = 37) also showed no statistically significant benefit in *EGFR-*mutant patients treated with durvalumab after cCRT [150]. Furthermore, real-world data including 16 *EGFR*-mutant patients in all 61 patients who received consolidation durvalumab found that the presence of an *EGFR*-mutation was the only independent predictive factor for unfavorable PFS (6.5 vs. 33.63 months in *EGFR* wild-type or unknown tumors; *P* < 0.001) [151]. In *EGFR/HER2*-mutant tumors, a significantly shorter DFS with durvalumab was also obtained (7.5 vs. NR; *P* = 0.04) compared with wild-type tumors [152]. Poor survival was independent of PD-L1 expression. For stage III *ALK* rearranged NSCLC patients, a retrospective analysis (*n* = 20) of patients with stage III ALK-rearranged NSCLC reached a similar conclusion [153]. Notably, the evidence against the use of ICIs consolidation therapy in this setting is also due to severe irAEs. Nearly 40% of patients experienced severe irAEs [150]. On the other hand, receiving tyrosine kinase inhibitors (TKIs) such as EGFR TKIs, during or after ICIs is also associated with increased toxicity. Up to 15% of patients receiving sequential osimertinib after ICIs treatment were reported to develop severe irAEs, and most of them required hospitalization [154]. When osimertinib is combined with durvalumab, the risk of interstitial lung disease may be higher [155].

Although larger prospective studies are urgently needed to confirm these findings, based on published data, in a recent ESMO consensus, over 90% of experts did not recommend the use of consolidation ICIs therapy after curative-intent CRT in *EGFR*-positive NSCLC [155]. As for alterations that might benefit from ICIs consolidation, as mentioned before, patients with a *KRAS* mutation (*n* = 26) may benefit from consolidation durvalumab (PFS not reached vs. 8.1 months in *EGFR*-mutant patients), similar to the metastatic setting [149]. In addition, durvalumab consolidation after cCRT significantly improves local–regional control in *KEAP1/NFE2L2* mutant NSCLC tumors (1-year regional failure of 62% vs. 25%, *P* = 0.021), which correlates with a chemoradiation-resistant phenotype, with a higher risk of locoregional failure [156]. These retrospective findings certainly add to the complexity of whether oncogenic addicted stage III NSCLC could derive benefits from ICIs consolidation, including some rare mutations, co-mutations, and other specifics. However, in summary, patients harboring driver mutations face an underwhelming prognosis with ICIs consolidation, with a hindered survival and unfavorable safety profile. Therefore, a better consolidative strategy for patients with *EGFR*-mutations and other oncogenic drivers is urgently needed. In this context, the role of targeted therapies is anticipated. The feasibility of RT combined with EGFR-TKIs has been reported in unresectable stage III *EGFR* mutation-positive NSCLC, although the sample size was limited [157, 158]. We should await the results of the randomized phase III LAURA trial, investigating the efficacy of adjuvant osimertinib after cCRT in patients with the most common *EGFR* sensitizing mutations (Ex19Del and L858R), which will provide evidence on the benefit of targeted therapy instead of ICIs [159]. In addition, another recruiting phase III multicenter study (NCT05170204) will evaluate the efficacy and safety of multiple therapies (alectinib, entrectinib, pralsetinib, and durvalumab) in cohorts of patients with *ALK*-positive, *ROS-1-*positive, or *RET* fusion-positive mutations in this setting [160]. Clearly, there is still a long way to go for this population, since genomic alterations are extraordinarily complex, and we cannot be satisfied with extrapolating data from the stage IV setting.

### Patient selection and biomarkers

Despite the increasing use of ICIs in patients with NSCLC, most patients do not benefit from such therapy. In addition, irAEs occurred in half of the patients, and the significant economic burden of ICIs cannot be ignored. Thus, it is imperative to develop suitable predictive biomarkers for efficacy and toxicity to select appropriate patients. In fact, multiple biomarkers have emerged as a research hotspot, which can be summarized into five categories: tumor itself, TME, liquid biopsies for circulating biomarkers, imaging biomarkers, and patient characteristics.

Previous studies have mainly focused on PD-L1 expression, tumor mutation burden (TMB), and microsatellite instability (MSI), but it is still far from the accurate screening of patients most likely to benefit. Tumoral PD-L1 immunohistochemistry was the first biomarker approved by the FDA and has been widely used to assist clinical decisions in the treatment of ICIs [8]. Multiple clinical trials have demonstrated that advanced NSCLC patients with relatively high tumoral PD-L1 expression tended to show improved responses to ICIs and longer survival [15, 161, 162]. Some studies have also reported the predictive role of soluble or exosomal PD-L1 expression [44, 163]. However, PD-L1 expression alone is not a perfect biomarker. First, several randomized trials disputed PD-L1 as a viable predictive biomarker for ICIs treatment, especially combined treatment [162]. For instance, the PEMBRO-RT trial revealed that PD-L1 negative patients had a much better response to iRT treatment than those positive [10]. Second, there is a lack of standardized PD-L1 assessment methods, as many variables exist in tumor sampling, testing, and assessment, as well as great heterogeneity across time and space in clinical, pathological, and TME characteristics. Notably, the assessed range, tumor, or both tumor and cells in the TME, and cut-off points remain to be unified. Third, tumoral PD-L1 expression is dynamic and influenced by multiple factors. In the non-metastatic setting, durvalumab showed PFS benefit in tumors with PD-L1 < 25% (HR, 0.59) in the PACIFIC trial [9]. However, post hoc analysis showed no OS benefit with durvalumab in tumors with PD-L1 < 1% (HR, 1.14; 95% CI: 0.71–1.84), although PFS benefit with durvalumab continued to be observed across all subgroups [50]. Consequently, durvalumab is not approved for tumors with PD-L1 < 1% in Europe, whereas it is approved irrespective of PD-L1 percentage in the US. The PACIFIC-R trial supported the feasibility of durvalumab in PD-L1-negative tumors, however, the median PFS was indeed shorter than PD-L1 ⩾1% tumors [51]. The abstract 8550 posted in ASCO 2022 demonstrated that patients with tumoral PD-L1 expression of < 1% had a significantly lower survival probability, compared to those of 1–50% and > 50% in patients with stage III unresectable NSCLC who received durvalumab post cCRT [135]. Low PD-L1 expression may originate from a lack of tumor infiltrating lymphocytes (TIL) and expression of other co-inhibitory checkpoints; therefore, strategies with dual checkpoint blockade consolidation are expected. The combinations from the COAST trial appeared to generate persistent benefits regardless of PD-L1 status; however, such reliability was limited by the number of patients available [66]. Meanwhile, BTCRC-LUN 16–081 reported significantly increased toxicity with the combination of nivolumab and ipilimumab [64]. Whether the downward trend of dual checkpoint blockade consolidation could be reversed will depend on the results of the CheckMate 73 L trial with a 1% stratification of PD-L1 expression.

Tissue TMB (tTMB), mostly determined by next-generation sequencing, is another leading candidate biomarker that has been widely evaluated in clinical trials, and the majority of the evidence comes from patients with lung cancer and melanoma [164]. Recently, the FDA approved pembrolizumab for patients with TMB ≥ 10 mutations/Mb in any tumor, following the results of the KEYNOTE-158 trial [165]. However, the reproducibility of such a cut-off ignited great controversy due to its arbitrariness and capriciousness [166]. There is no consensus on gene panel size and methodology for measurement [8]. Another major concern is that TMB assessment and bioinformatics interpretation vary across different cancer types as well as subgroups of different characteristics from a single type of cancer [167, 168]. Thus, the accuracy of blood TMB (bTMB) is worthy of verification. The role of TMB in terms of RT or iRT is uncertain. Generally, the predictive value of TMB alone is limited. One study evaluated the incorporation of TMB and PD-L1 expression into multivariable predictive models and demonstrated a greater predictive power [169]. Mismatch repair deficiency (dMMR) and MSI have also been considered to earn the competence in predicting ICIs efficacy [170, 171]. Likewise, clinical application is difficult because of the lack of higher levels of evidence and difficulties in detection, especially with repeated biopsy sampling for dynamic monitoring.

TILs, such as CTLs, reflect the TME more directly, and adequate lymphocyte infiltration is necessary for ICIs to exert anti-tumor effects. A small population of progenitor exhausted cells among exhausted CD8 + TILs mediates long-term tumor control and responses to anti-PD-1 therapy [172]. It has been found that a TIL density of over 10% could predict better survival benefits from ICIs [173]. In terms of RT, CD8 + TIL density increased after cCRT and higher density post-cCRT predicted favorable clinical outcomes [174]. The characteristics of TILs, including their composition, organization, density, and functional state, may jointly predict responses to iRT. Of note, similar to tumoral PD-L1 expression, difficulties in detection resulting from limited histologic material from the biopsy restrict direct evaluation of TIL. The predictive roles of Tregs, MDSCs, and some immunoregulatory pathways, such as CD28/B7 and TIM-3, which together constitute and regulate the TME, are also being studied [175]. In addition, multiple patient-specific gene expression profiles (GEPs) characterizing the TME have been investigated, such as targeting T-cell inflammation, antigen processing and presentation, and immunosuppressive molecules. An 18-gene profile of T cell inflammation demonstrated strong correlations with clinical outcomes in a wide variety of solid tumors treated with pembrolizumab [176]. As for RT, with further exploration of radiobiology, individualized RT with dose adjustment based on genomes is also being developed [177]. Although not nearly enough, these multigene signatures characterizing radiosensitivity, TME, and immune-related mechanisms hold tremendous potential to predict RT, ICIs, and combination therapy strategies. In addition, some specific oncogenic alterations in pivotal signaling pathways have been reported to predict responses to ICIs, such as Wnt/β-catenin, PTEN, PI3K-AKT, EGFR, c-Met, ALK, and KRAS [175].

Difficulties in detection include the use of biomarkers for restrictions in lung biopsy. Liquid biopsy, developed to solve pain, has attracted great attention and has become increasingly popular owing to its feasibility and ease of operation. Of note, it has brought about a dynamic evaluation of the responses of possibility. In principle, any tumor-derived material circulating in peripheral blood can be analyzed, including ctDNA, circulating tumor cells (CTCs), circulating tumor RNA (ctRNA), tumor endothelial cells (TRCs), and exosomes. Among these, ctDNA derived from tumor cells is the most commonly used modality [139]. The prognostic value of MRD detection in patients with LA-NSCLC and the role of dynamic ctDNA in facilitating personalized consolidation ICIs strategies have been elaborated previously, which is of great potential from our point of view. However, factors such as assay type, amount of ctDNA released, and technical and biological background can all impact ctDNA MRD results. Therefore, the clinical utility of ctDNA MRD for the personalized treatment of solid tumors, including NSCLC, remains to be fully established. Another alternative marker from non-invasive detection, CTCs, may be used to evaluate dynamic variations in immune checkpoint expression during treatment [178]. Concordance between tumoral PD-L1 expression and CTCs has been reported to be as high as 93% in advanced NSCLC. Nevertheless, a common limitation of biomarkers for LA-NSCLC is that the low tumor burden in already-treated localized diseases would impact the isolation of CTCs. In addition, peripheral blood cells and lymphocytes are thought to indirectly reflect immune responses; however, they are not clinically applicable. ctRNAs with the advantages of stability have also been recognized as potential biomarkers, which need to be validated in larger cohorts. It is conceivable that dynamic liquid biopsy will guide the duration of ICIs or combined treatment in the future, with validation in larger prospective trials. At present, including in LA-NSCLC, dynamic ctDNA is undoubtedly the most promising for clinical applications.

Imaging information is the standard assessment of treatment efficacy and image-guided RT, including CT, MR, and PET/CT was the basis for precision RT. PET/CT, which integrates metabolic and anatomical information and is also called functional imaging, naturally reflects the TME. Several studies have explored the possible correlation between [18]F-FDG-uptake and existing ICIs sensitivity markers, such as PD-L1 and CD8 + TILs in tumor tissues [179, 180]. Our previous study also indicated a correlation between [18]F-RGD uptake and tumor PD-L1 expression [181]. Notably, benefiting from its non-invasiveness, PET/CT may also reflect dynamic changes in the TME under ICIs. In addition, a predictive model based on high-throughput image characteristics, namely radiomics, is a promising method. A recent study indicated that tumor radiomics of pretreatment CT images was a prognostic factor for outcomes in patients with stage III unresectable NSCLC treated with CRT followed by durvalumab or CRT alone [182].

It is evident that a single biomarker cannot serve a powerful and comprehensive predictive function, even beyond PD-L1 expression. However, a variety of biomarkers have emerged as complementary predictors of response, including combining two or more biomarkers to increase accuracy. It is foreseeable that the integration of multi-omics information, including patient characteristics, imaging, pathology, peripheral blood, and genomic information, as a predictive tool to guide comprehensive treatment, will be the direction of development. Machine learning that integrates multimodal features can be a prospective approach for predicting treatment response [183]. Meanwhile, non-invasive and real-time monitoring for prediction requires technological advances.

### Novel combination strategies

Although the combined treatment improved responses in irradiated and non-irradiated tumors, resistance was also common. Owing to the distinct mechanisms of action, dual or triple checkpoint blockade is expected to work synergistically. Except for the molecules mentioned before, there are also some other co-inhibitory receptors including Lag-3, Tim-3 and B7-H3 are being explored [184, 185]. Costimulatory molecules such as the CD122 agonists NKTR-214, 4-1BB, OX-40, GITR, TLR9, and STING have also been exploited to enhance anti-tumor activity [186]. In practice, these agents are usually first tested in advanced NSCLC, and only successful applications will help them move forward to LA-NSCLC. Notably, bispecific antibodies (BsAbs) targeting two different checkpoints are emerging, which have significant advantages over combination therapy using two different mAbs, including reduced development and therapeutic costs, higher binding specificity and obligate effects [187].

Antiangiogenic agents targeting the vascular endothelial growth factor and its receptor are promising agents in combination with ICIs and RT. It is assumed that anti-angiogenic agents promote the trafficking of immune effector cells, drive DC maturation, reduce MDSCs and Tregs, and limit hypoxia partly via vessel re-normalization, thereby functioning as ideal partners for ICIs and superior radiosensitizers [186]. A quadruple combination regimen of antiangiogenic atezolizumab and carboplatin–paclitaxel chemotherapy in non-squamous NSCLC (ABCP regimen) has achieved great success in advanced non-squamous NSCLC [188]. Based on this clinical and preclinical evidence, RT is expected to further potentiate the anti-tumor effects of ICI and angiogenesis dual blockade. Such a triple combination therapy for LA-NSCLC is a promising direction for future research. In contrast, molecular-targeted agents combined with ICIs have been hampered by severe toxicities, and their addition to the treatment of LA-NSCLC seems a long way off.

**Fig. 4:**
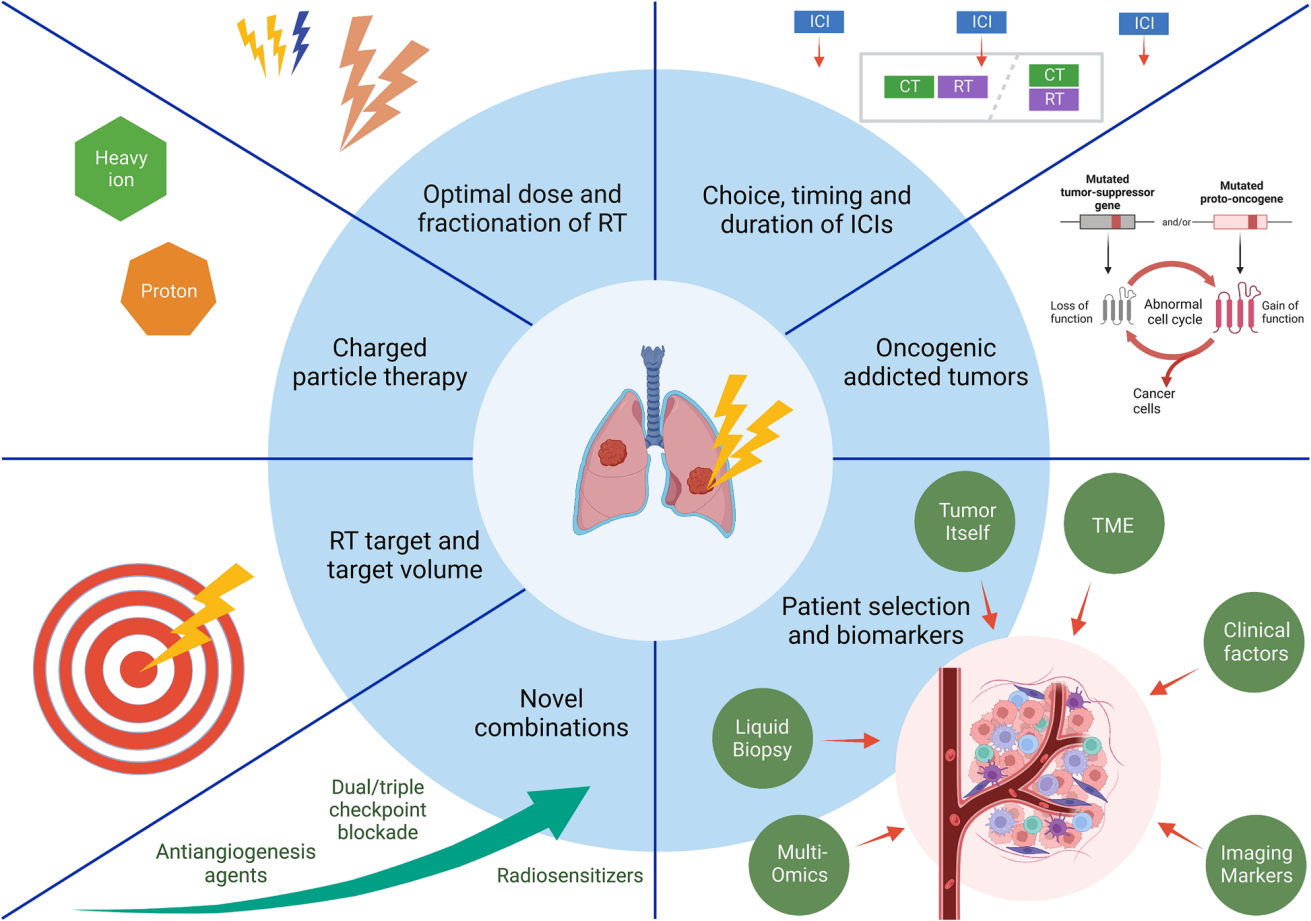
Challenges, strategies, and auspicious orientations of iRT in LA-NSCLC. The existing challenges of iRT in LA-NSCLC include optimal dose and fractionation of RT, optimal RT target and target volume, application of charged particle therapy, optimal timing and duration of ICIs, iRT in oncogenic addicted tumors, optimal biomarkers and novel combinations, which are also strategies and auspicious orientations. Abbreviations: *RT* Radiotherapy, *ICIs* Immune checkpoint inhibitors, *iRT* ICIs combined with RT

Hypoxia is an important obstacle contributing to resistance to RT and immunosuppression, facilitating tumor recurrence and metastasis [189]. Radiosensitizers can sensitize hypoxic or radioresistant tumors to RT. With advancements in nanotechnology, the application of nanoparticles to overcome resistance to RT and enhance RT efficacy against tumors has become an area of intense research, as radiosensitizers are usually loaded or engineered into nanocarriers for delivery [190]. For example, a multifunctional nanoprobe, based on quantum dots emitting in the near-infrared IIb window, can effectively aggregate at the tumor site to precisely image the tumor region with high resolution, promote the radio-sensitivity and immunogenicity of cancer cells, and relieve intratumoral hypoxia to enhance RT-based therapy strategies [191]. Nanoprode-mediated immunogenic RT can exert a more intense and enduring anti-tumor effect when combined with immunotherapy. Several nanoscale metal–organic frameworks also notably enhance the effects of ionizing radiation, serving as a powerful adjuvant therapy to synergize with iRT, termed RT–radiodynamic therapy [192, 193]. Although more clinical trials are needed, novel materials, especially nanomaterials, are rapidly emerging as the frontier of cancer treatment and have immense potential. We summarize the challenges, strategies and auspicious orientations of iRT in LA-NSCLC in Fig. 4.

## Conclusions and future perspectives

Definitive RT plays a major role as a local therapy in unresectable LA-NSCLC. In addition, RT promotes tumor regression via ICD, whereas the recruitment of immunosuppressive cell populations and exacerbation of tumor hypoxia can engender a radioresistant phenotype. Boosting these positive effects while mitigating undesirable effects can be exploited to improve clinical responses. Combining ICIs with RT is a proven and potential synergy through the mutually beneficial remodeling of the TME, which is expected to overcome resistance to RT and ICIs and augment the abscopal effect of RT and the immune memory effect. However, issues remain regarding the rational dosage and fractionation of RT and timing of combined ICIs, relying on further understanding of the paradoxical effects of RT on TME and direct comparison both in preclinical and clinical studies. Notably, hypofractionated regimens and SBRT combined with LDI are superior. In addition, TME heterogeneity within and between patients directly influences the effects of combined therapy. Effective biomarkers guiding patient selection assist precision therapy, and the integration of multi-omics information for prediction is the way forward. Finally, novel RT technologies and innovative combination strategies are promising approaches to exploit the untapped therapeutic potential. In general, iRT is a proven and potential strategy in LA-NSCLC, with multiple promising approaches to further improve the efficacy.

## Data Availability

Not appliable.

## Abbreviations

ICIs: Immune checkpoint inhibitors
RT: Radiotherapy
LA-NSCLC: Locally advanced non-small cell lung cancer
iRT: RT combined with ICIs
NSCLC: Non-small cell lung cancer
LA: Locally advanced
DNA: Deoxyribonucleic acid
cCRT: Concurrent chemoradiotherapy
SoC: Standard of care
OS: Overall survival
ORR: Objective response rate
TME: Tumor microenvironment
SBRT: Stereotactic body radiation therapy
PD-1: Programmed cell death 1
PD-L1: Programmed cell death-ligand 1
CTLA-4: Cytotoxic T-lymphocyte associated antigen 4
PFS: Progression-free survival
DAMPs: Damage-associated molecular patterns
HMGB1: High mobility group box 1
ATP: Adenosine triphosphate
ICD: Immunogenic cell death
ROS: Reactive oxygen species
TAAs: Tumor-associated antigens
dsDNA: Double-stranded DNA
DCs: Dendritic cells
APCs: Antigen-presenting cells
CTLs: Cytotoxic lymphocytes
PRRs: Pattern recognition receptors
TLR: Toll-like receptor
MHC: Major histocompatibility complex
TCR: T-cell receptor
GMP: Cyclic guanosine monophosphate
AMP: Adenosine monophosphate
cGAS: Cyclic GMP-AMP synthase
STING: Stimulator of interferon genes
mtDNA: Mitochondrial DNA
AIM2: Absent in melanoma 2
IFI16: Interferon-inducible protein 16
IRF: Interferon regulatory factor
TAMs: Tumor-associated macrophages
PMNs: Polymorphonuclear neutrophils
MDSCs: Myeloid-derived suppressor cells
CAFs: Cancer-associated fibroblasts
SDF-1: Stromal-derived factor 1
CSF-1: Colony stimulating factor-1
Tregs: The regulatory T cells
HIF-1α: Hypoxia-inducible factor 1-α
HR: Hazard ratio
sCRT: Sequential chemoradiotherapy
CRT: Chemoradiotherapy
TRAEs: Treatment-related adverse events
AEs: Adverse events
TDLNs: Tumor-draining lymph nodes
TMDD: Time to metastatic disease or death
DCR: Disease control rate
PARP: Poly adenosine diphosphate-ribose polymerase
PR: Partial response
PET/CT: Positron emission tomography/computed tomography
irAES: Immune-related adverse events
CIP: ICIs-related pneumonitis
HyperRT: Hyperfractionation RT
HypoRT: Hypofractionated RT
SABR: Stereotactic ablative radiotherapy
Trex1: Three prime repair exonuclease 1
ENI: Elective nodal irradiation
LDI: Low dose irradiation
CIRT: Carbon-ion RT
ctDNA: Circulating tumor DNA
MRD: Minimal residual disease
NGS: Next-generation sequencing
TKIs: Tyrosine kinase inhibitors
TMB: Tumor mutation burden (MSI)
MSI: Microsatellite instability
TIL: Tumor infiltrating lymphocytes
tTMB: Tissue TMB
bTMB: Blood TMB
dMMR: Mismatch repair deficiency
GEPs: Gene expression profiles
CTCs: Circulating tumor cells
ctRNA: Circulating tumor RNA
TRCs: Tumor endothelial cells
LDH: Lactate dehydrogenase
LIPI: Lung immune prognostic index
NLR: Neutrophil to lymphocyte ratio
PLR: Platelet to lymphocyte ratio
LMR: Lymphocyte to monocyte ratio
